# Enhanced ordering reduces electric susceptibility of liquids confined to graphene slit pores

**DOI:** 10.1038/srep27406

**Published:** 2016-06-06

**Authors:** Jeronimo Terrones, Patrick J. Kiley, James A. Elliott

**Affiliations:** 1 Department of Materials Science and Metallurgy, University of Cambridge, 27 Charles Babbage Road, Cambridge CB3 0FS, United Kingdom

## Abstract

The behaviours of a range of polar and non-polar organic liquids (acetone, ethanol, methanol, N-methyl-2-pyrrolidone (NMP), carbon tetrachloride and water) confined to 2D graphene nanochannels with thicknesses in the range of 4.5 Å to 40 Å were studied using classical molecular dynamics and hybrid density functional theory. All liquids were found to organise spontaneously into ordered layers parallel to the confining surfaces, with those containing polar molecules having their electric dipoles aligned parallel to such surfaces. In particular, monolayers of NMP showed remarkable in-plane ordering and low molecular mobility, suggesting the existence of a previously unknown 2D solid-like phase. Calculations for polar liquids showed dramatically reduced static permittivities normal to the confining surfaces; these changes are expected to improve electron tunnelling across the liquid films, modifying the DC electrical properties of immersed assemblies of carbon nanomaterials.

The study of fluids confined to nanoscopic channels is of paramount importance in a wide variety of fields, ranging from soil mechanics to lubrication and cell biology^1^,^2^,^3^,^4^,^5^, but in this paper we particularly focus on the structure and dielectric properties of organic liquids confined to nanoscopic graphene slit pores. Our interest in this configuration, in which a thin film of liquid is “sandwiched” between graphitic walls, arises from its resemblance to an immersed junction between two bundles of carbon nanotubes (CNT), which is described in detail in the methods section. Interbundle junctions play a significant role in determining the electrical properties of macroscopic assemblies of CNTs^6^ and the results of the current study, which show that liquids organise spontaneously into ordered layers parallel to the confining surfaces, help to understand better a series of electro-structural phenomena recently discovered in CNT fibres immersed in organic liquids^7^,^8^,^9^. Furthermore, the recent possible discovery by Algara-Siller *et al.* of “square-ice” (a new two-dimensional solid-like phase of water), stable at ambient conditions inside graphene slit pores, adds further interest to this configuration, since other organic liquids may also present previously unknown 2D solid-like structures^10^.

Experimental work has revealed confinement-induced organization in solvent molecules^2^,^11^,^12^,^13^,^14^. Israelachvili *et al.* used a surface force apparatus (SFA) to confine solvent molecules between two mica plates with a spacing of only a few nanometres and measured the force of attraction as a function of their separation^2^,^11^,^12^. For relatively rigid molecules, this force was found to be oscillatory with distance at small plate separations, which was explained by the formation of layers, but rapidly approached the value for bulk liquids at plate separations between 4 and 10 molecular radii. Further studies by Christenson indicated that this phenomenon was less likely to occur with more flexible molecules^13^. Studies with a transverse dynamic force microscope by Antognozzi *et al.* showed that the oscillatory force period for water was between 0.24 and 0.29 nm, consistent with the diameter of water molecules^15^. SFA experiments measuring shear forces found that at particular separations, the mica plates would not slide until a threshold shear stress was reached. This behaviour, which is uncharacteristic for bulk liquids, was interpreted as the formation of “solid-like” structures at such separations^2^,^12^,^14^,^16^. These experimental results have been corroborated by a variety of simulation studies, mainly performed for water^1^,^17^,^18^,^19^,^20^,^21^. In particular, using simulations with the classical SPC potential for water, Martí *et al.* predicted an increased dielectric constant for water confined to a graphene slit pore^20^,^21^. We will further assess their conclusions later in the results section of this paper.

In this work, we perform both classical molecular dynamics (MD) and hybrid density functional theory (DFT) calculations to explore the behaviour of acetone, ethanol, methanol, *N*-methyl-2-pyrrolidone (NMP), carbon tetrachloride, and water confined to graphene slit pores in the 4.5 Å to 40 Å thickness range. All our simulations were carried at room temperature (298 K) and standard atmospheric pressure (1 bar). We report on the effects of confinement on the transverse as well as in-plane structure of the liquids, and the possibility of new solid-like phases. The effects of confinement-induced order in the dielectric properties of liquids are studied by applying a variety of constant electric fields in the transverse direction (*i.e.* normal to the graphene sheets). Finally, we discuss how the observed changes in electric susceptibility may affect the electrical properties of immersed carbon nanotube fibres and similar nanocarbon-based hierarchically-structured materials.

## Results

**Figure 1:**
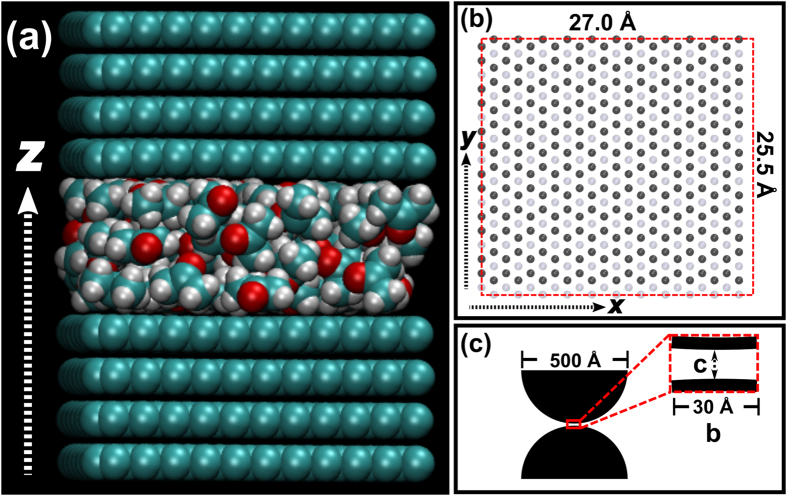
(**a**) Snapshot of a typical simulation cell showing the 8 graphene sheets and the “sandwiched” liquid film (acetone in this case). (**b**) “Top view” of a simulation cell showing the AB-stacking of graphene sheets and the periodic boundaries (dashed square). (**c**) Schematic of a junction between two bundles of carbon nanotubes; our model emulates the conditions in the magnified section.

Confinement of solvents by graphene slit pores was simulated according to the general scheme shown in Fig. 1(a,b). Briefly, a cavity was introduced between two adjacent layers of an A-B stacked graphite supercell and filled with liquid. Atmospheric pressure was maintained by regulating the separation between the graphite blocks. To prevent any ambiguity, from here onwards we will refer to the entirety of the confined liquid as a “film”; the terms “layer” and “monolayer” will be reserved to describe the sub-structure within the film, and “sheet” will be used for the graphene only. Figure 1(c) presents a schematic of the cross-section of an open junction between two bundles of CNTs. Taking a look at the magnified region (curvature drawn to scale) it can be seen that the narrowest part of the junction appears locally flat at the scale of our simulation cell, justifying the use of our model to investigate the properties of a CNT junction. Please refer to the methods section for a more detailed description of our model, its validity, and the simulation techniques used for this work.

### Transverse solvent ordering

**Figure 2:**
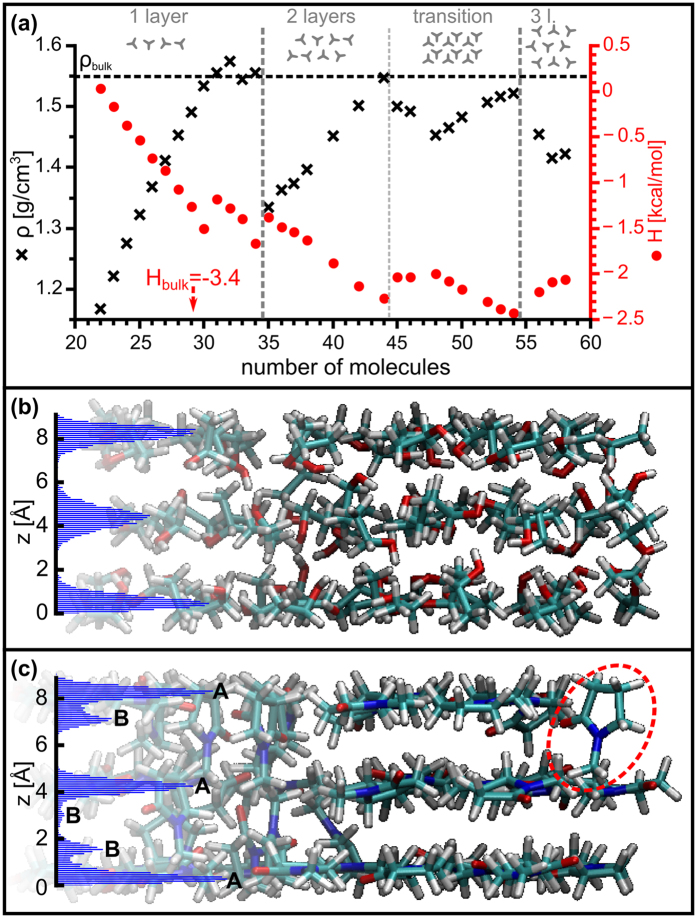
Transverse ordering of liquids in the graphene slit pore: (**a**) plot of density (diagonal crosses, left axis) and enthalpy (circles, right axis) as a function of the number of molecules, n_m_, for CCl_4_; schematics on the top illustrate the different layered structures observed. (**b**) z-distribution of the geometric centres of ethanol molecules in a cell containing n_m_ = 88 molecules. (**c**) Same as (**b**) but for NMP with n_m_ = 54.

All studied liquids demonstrated a characteristic transverse layering resulting from confinement in the *z*-direction. Figure 2(a) shows changes in density, *ρ*, and enthalpy, *H*, of simulated CCl_4_ as a function of the number of molecules, *n*_*m*_. It can be seen that both quantities tend to the values calculated for bulk CCl_4_ as *n*_*m*_ increases. The density plot shows very sharp drops, marked with vertical dashed lines in the figure, which correspond with changes in the liquid’s layer structure, schematically depicted in the top part of the figure. CCl_4_ is thus seen to progress from a single monolayer to two disordered monolayers, then to two close packed (slightly thicker) monolayers, and finally to three disordered monolayers. Additional data for other solvents (acetone, ethanol, methanol, NMP, and water) are given in in supplementary Figures S3 and S4, and demonstrate that all of them exhibit qualitatively similar behaviours.

The organization of the trapped liquid molecules into layers is visually evident when observing rendered snapshots from any simulation; Fig. 2(b,c) show such ordered structures for tri-layered configurations of ethanol and NMP, respectively, with superimposed plots of the transverse distribution of the geometric centres of molecules. Correspondingly similar distribution profiles were obtained for all other liquids and different molecule numbers (see SI. 3.2). In all cases, the sharpest peaks, indicating the most ordered layers, are those in contact with the graphene surfaces. Structural order is gradually lost towards the centre of the slit pore, especially in the case of thicker liquid films (larger *n*_*m*_). The degree of ordering and its rate of decay with distance from the graphene walls depend on the particular liquid being simulated. Of all the liquids studied, ethanol displays qualitatively the most defined layering, showing 8 very distinct layers for the thickest (*n*_*m*_ = 235, 33.7 Å) film (Figure S5(c)). On the other hand, the smallest molecule, water, shows barely discernible layers towards the centre of its thickest (*n*_*m*_ = 235, 22.7 Å) film (Figure S5(e)).

The behaviour of NMP is slightly more complicated than that of all other liquids studied; in Fig. 2(c), its transverse profile shows 6 peaks grouped in pairs, instead of the 3 peaks expected from a tri-layered structure. Each pair consists of a main peak and a smaller subsidiary peak, labelled respectively *A* and *B*, with their centres separated by 1.1 ± 0.1 Å. These double-peak profiles, which tend to have a mirror plane at the centre of the slit pore (Figure S6), are caused by NMP molecules assuming one of two orientations within the monolayer: the majority of molecules keeping their long axes parallel to the graphene sheets (peak *A*) and a few ones assuming the “standing” position indicated by a red dashed ellipse in Fig. 2(c) (peak *B*). Perhaps due to the more symmetrical shape of their molecules, this effect was not observed in the other liquids. The average separations between two consecutive monolayers (the interlayer distances *d*_*il*_) are listed in Table 1, along with other structural parameters. For the case of NMP, *d*_*il*_ was measured as the average distance between consecutive *A* peaks.

### In-plane solvent ordering

**Figure 3:**
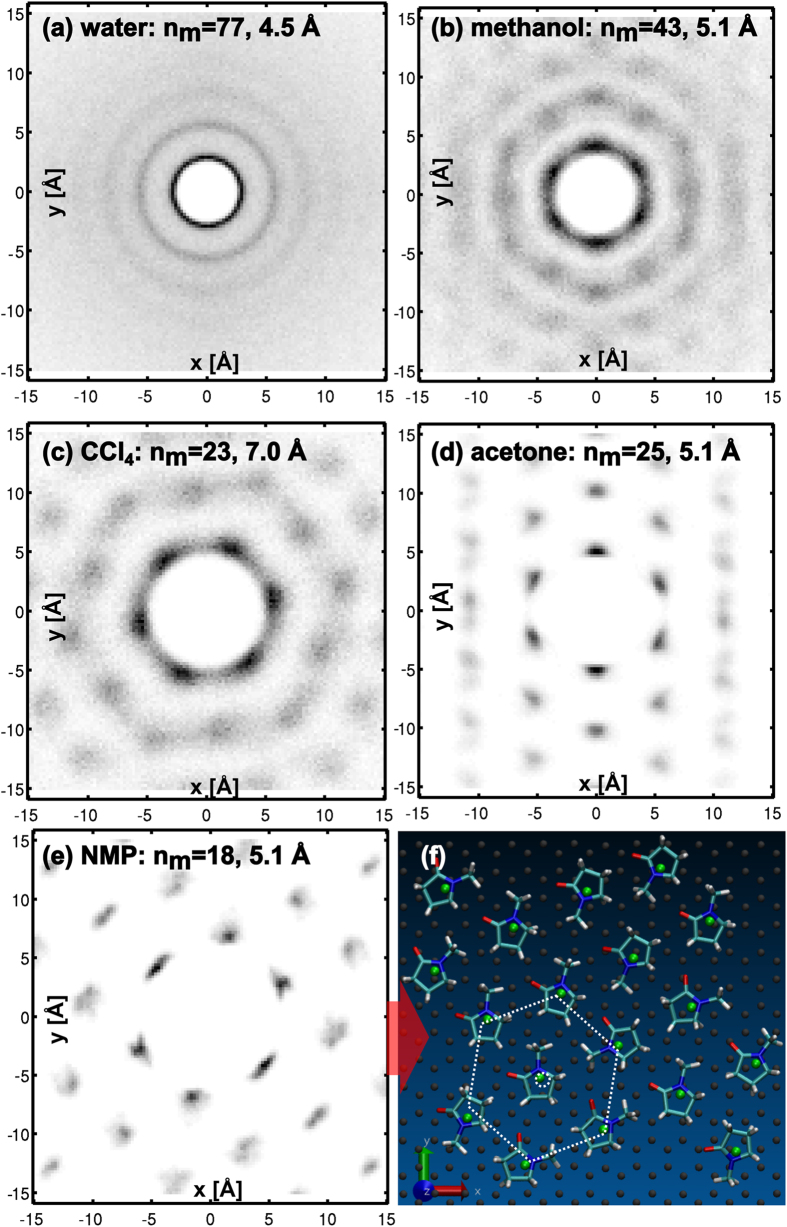
(**a–e**) Pair distribution functions of the geometric centres of liquid molecules in monolayers of water (**a**), methanol (**b**), carbon tetrachloride (**c**), acetone (**d**), and NMP (**e**). (**f**) Snapshot of a simulation cell of a monolayer of NMP showing the graphene lattice (grey spheres) and the NMP molecules; the geometric centres of the NMP molecules are indicated with green spheres.

Figure 3(a–e) shows 2D pair distribution functions (PDF) in *x-y* plane for the geometric centres of, respectively, water, methanol, CCl_4_ acetone, and NMP molecules in their thinnest films, where only a monolayer of liquid separates the graphene sheets. In these representations, a molecule is supposed to be centred at the origin and the darkest areas then represent the zones of highest probability to find another molecule. The degree of ordering within the monolayers varied widely depending on the molecule studied: from water (Fig. 3(a)), which shows a ring pattern typical of the short-range ordering of bulk liquids, all the way to acetone and NMP (Fig. 3(d,e) respectively), which show a very clear ordered structure (2D triangular lattice) more typical of the long-range order in a crystalline solid or a liquid crystal. For other molecules the ordering seems to be at an intermediate point between the extremes of water an NMP. With the exception of water, all molecules show short-range 2D hexagonal order that decays rapidly away from the origin. The high degree of ordering in the monolayer of NMP can be further appreciated in the simulation snapshot shown in Fig. 3(f); there, it is clear that NMP molecules form a hexagonal pattern, with their long axes parallel to the graphene sheets, and their oxygen atoms (coloured red) seemingly pointing in a preferential direction. A similar structure was observed for acetone (Figure S10). The average distance from the centre of the molecules to those of their nearest neighbours in the monolayer (measured from the PDFs), *d*_*nn*_, and their effective molecular Lennard-Jones diameters in the bulk (from ref. 22), *D*_*LJ*_, are listed in Table 1. The very small difference between these values gives a measure of confidence of our simulation methods.

PDFs of individual monolayers within thicker liquid films were also calculated (Figures S8 and S9). These showed that the order in the layers in contact with graphene is not as good as in the case monolayers (compare Fig. 3(e) here with Figure S8(b1)) and, analogously to transverse ordering, it further decreases towards the centre of the films. For the case of films containing 2 layers only, the distribution of molecules in one layer with respect to the other one was measured (Figure S9). The layer correlation functions showed that, in all cases with the exception of water, molecules in one layer avoid adopting positions directly on top of those in the adjacent layer (reminiscent of A-B stacking).

**Figure 4:**
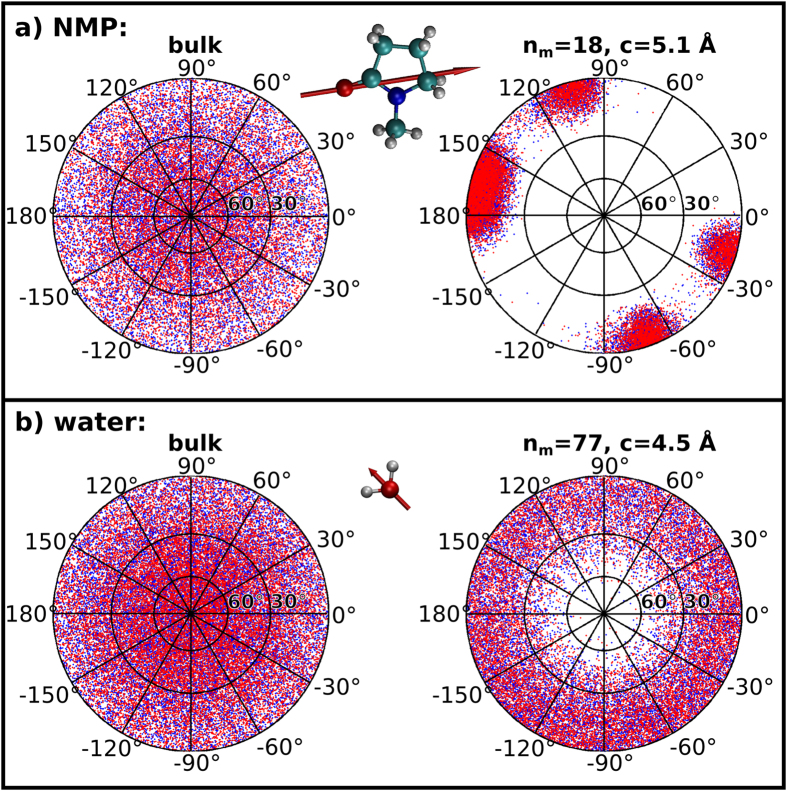
Orientation of molecular dipoles of NMP (**a**) and water (**b**) in the bulk (left) and in a monolayer (right); the red arrow in the molecules indicates the orientation of the molecular dipoles.

Figures 2(c) and 3(f), and S10 (as well as the considerable difference between *d*_*il*_ and *d*_*nn*_ for NMP) already suggest that NMP and acetone molecules assume preferred orientations within individual monolayers. Being interested in the dielectric properties of the liquid films, we have further studied the orientation of these and the other polar molecules (*i.e.* all but CCl_4_) by analysing the orientation of their permanent electric dipole moments, 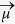, during the simulations. Figure 4 presents stereographic plots (see SI. 6 for calculation details and further plots) of the orientation of the molecular dipoles (depicted as red arrows in the figure) of NMP (a) and water (b) both in the bulk and in the monolayer; red and blue dots represent positive and negative elevation angles respectively. In this figure it is clear that, while being randomly oriented in the bulk liquid, the molecular dipoles of both NMP and water assume a series of preferred elevation orientations (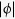 < 30°) in the monolayer, with NMP further assuming a series of preferred azimuthal orientations (−90° < *θ* < −60°, −30° < *θ* < 0°, 90° < *θ* < 120°, 150° < *θ* < 180°). Similarly to these examples, the dipoles of all other confined liquids assumed preferential elevation angles, tending to lay parallel to the confining graphene surfaces (*i.e.* assuming small values of 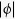 or, equivalently, of |*μ*_*z*_|) even in films thicker than a single monolayer (Figures S12–S14). Strong orientation of dipoles in the azimuthal direction was observed for NMP and acetone only (Fig. 4(a) and Figure S12(a,d)). Figure 2(c) shows that, in NMP films composed of more than one monolayer, a minority of molecules tend to assume an alternative “standing” orientation responsible for the B peaks in the figure. In fact, this “standing” orientation, for which 40° < 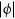 < 60°, becomes the predominant one for a particular series of cases when the NMP film is transitioning from a single monolayer to two (thickness ~7.3 Å, see SI. 7). After this transition the conventional “flat” orientation of NMP dipoles returned to be the principal one for the remainder of simulations.

The existence of preferred orientations for the molecular dipole moments of liquids confined to slit pores indicates that electric fields across the pore will have to work against “orientational forces” in order to align the molecules, which, as will be showed later dramatically affects the polar liquids’ orientational polarizabilities and, thus, their dielectric constants.

### Solid-like phases of 2D solvent films

The recent alleged experimental discovery of “square ice” inside a graphene pore at room temperature^10^, has elicited curiosity about whether other liquids confined by graphene may also form previously unknown 2D solid or solid-like structures. The high degree of order observed in the monolayers of acetone and NMP suggests that these two solvents might have transitioned to solid-like phases. In addition to PDFs, we have studied the mean square displacement (MSD) of molecules as a function of time to judge how solid- or liquid-like a particular configuration is with respect to the bulk. Table 2 compares the slopes of MSD vs. time plots for various simulations including those of bulk liquids, monolayers, and “thick” solvent films (~8 layers). Since, in monolayers, solvent molecules are confined in the *z*-direction, all measurements exclude *z*-displacement for a better comparison; the final column lists the ratio of the slopes of bulk solvents to those in a monolayer. It can be seen that the most dramatic change occurs for NMP, for which its molecules are approximately 600 times less mobile in the monolayer than in the bulk, strongly suggesting a transition into a solid-like phase. Interestingly, the in-plane mobilities of CCl_4_ and water do not seem to be significantly affected by confinement. The mobilities of the remaining liquids were reduced by around an order of magnitude, with acetone being the second most affected liquid. The change in acetone, weaker than in NMP, seems more compatible with a liquid-crystalline behaviour.

In the bulk, NMP solidifies at −24 °C (249 K); its crystalline solid form (studied at 168 K^23^) has a monoclinic structure with lattice parameters *a* = 6.221 Å, *b* = 12.076 Å, *c* = 7.529 Å, and β = 111.03˚, and four molecules in each unit cell. The hexagonal structure we observed for the monolayer of NMP (Fig. 3(e,f)) does not match with the bulk structure, which suggests that it may be an entirely new structure stable at room temperature. However, it is also possible that the aspect ratio and the fixed number of molecules per unit area, artificially imposed by the periodic boundary conditions into the simulation cell, are favouring the hexagonal structure. As a precaution against this, we ran a series of simulations with a cell with aspect ratio and molecule density closer to that expected for a slice of bulk NMP (see SI. 8). A hexagonal pattern, albeit oriented in a different direction, is still observed. At this point, we can conclude that our results suggest strongly that NMP may adopt a novel structure under confinement; however more experimental and theoretical work (currently underway) is required to fully verify this prediction.

Despite NMP showing a solid-like behaviour, no evidence of the occurrence of “square ice” or any other solid-like phase of water was found in the simulations of water monolayers: the PDF shown in Fig. 3(a) and the slope of the MSD vs. time curve from Table 2 suggest a bulk-like behaviour for water. This discrepancy may be due to an increased transverse compression of the water monolayer due to adhesion forces between the graphene layers in the experimental work of Algara-Siller *et al.* In their experiment, small droplets of water were trapped in small cavities between two graphene sheets with most of the sheets being in contact with one another–enforced in their MD simulations mainly by fixing the separation of the graphene layers^10^, something avoided by our piston-style pressure control. Furthermore, considering the ongoing controversy regarding the experimental results of Algara-Siller *et al.*, Zhou *et al.* have argued that the alleged “square ice” may actually be NaCl nanoplatelets arising from a contaminated sample^24^, to which Algara-Siller *et al.* have replied that, while they lack experimental data to fully disprove contamination, their samples were at never in contact with NaCl or any other salts^25^. Nevertheless, our simulations carried out with piston-style pressure control indicate the absence of “square ice”. The fact that, using the same method, a solid-like phase is still found for NMP suggests that the 2D solid-like configuration of NMP may be even more stable than that of water, which appears to require a transverse compressive force.

### Effect of confinement on the static dielectric properties of liquids

**Figure 5:**
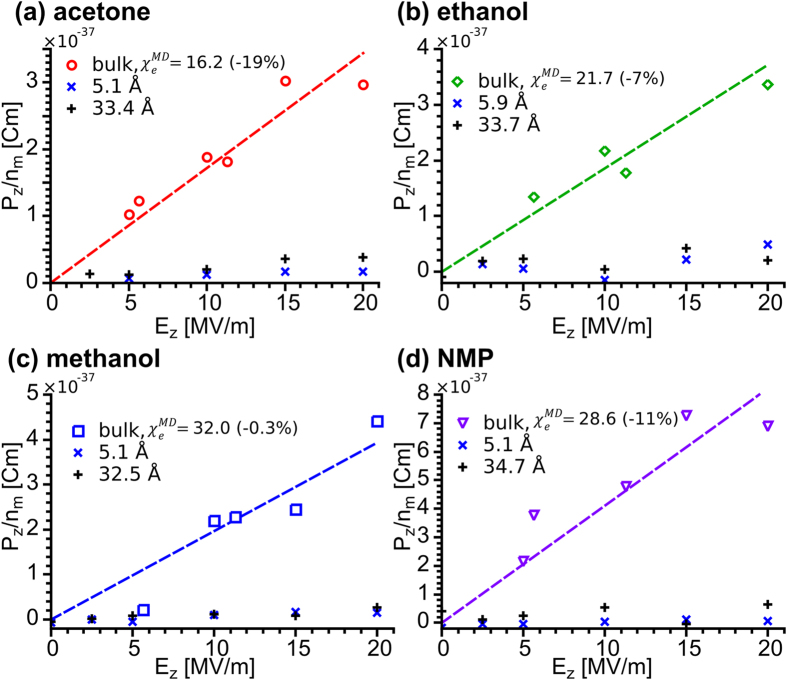
Normalized transverse component of total dipole moment, P_z_/n_m_, as a function of transverse electric field, E_z_, for: (**a**) acetone, (**b**) ethanol, (**c**) methanol, and (**d**) NMP. Lines correspond to slope fits to the data points of the same colour.

Figure 5 shows the normalized transverse component of total dipole moment, *P*_*z*_*/n*_*m*_, as a function of transverse electric field, *E*_*z*_, applied to different simulation cells of: (a) acetone, (b) ethanol, (c) methanol, and (d) NMP. Here, the total dipole moment, 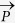 = (*P*_*x*_, *P*_*y*_, *P*_*z*_), is the vector sum of all the dipoles of the individual molecules in the cell (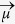). It is clear that the ability of the electric fields to induce a dipole in the *z*-direction is significantly hampered by the confining surfaces. The slopes of the *P*_*z*_*/n*_*m*_ *vs. E*_*z*_ plots correspond with the average static molecular polarizability of the liquids in the transverse direction, 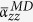, and can be used to estimate the static electric susceptibility, 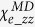, (see SI. 9.1) from


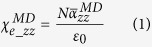


where *N* is the liquid’s number density and *ε*_0_ is the permittivity of free space. The subscript *zz* indicates that we are dealing with the diagonal elements in the *z*-direction of the 3 × 3 polarizability and susceptibility tensors; in the case of bulk liquids, which are isotropic, the quantities are scalar and the indices can be dropped. The superscript *MD* indicates that the calculations come from our MD simulations and, thus, disregard the effects of electronic polarizability (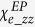, to be discussed later).

Equation (1) was used first to calculate the susceptibilities of bulk liquids and yielded the results shown in the figure, with the values in parentheses indicating the deviation from the values from literature^26^. For water, not shown in the figure, the obtained value was 93 (about 17% higher than accepted experimental bulk value at 20 °C). Considering the approximations of our method, the observed discrepancies are not unreasonable, especially as we will be comparing ratios of susceptibility in bulk liquid to confined solvent films.

We calculated 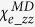 for a series of selected films of different liquids, and the results are summarized in Table 3. All the calculated values are at least one order of magnitude smaller than those calculated for the bulk liquids. The strongest effect of confinement is seen for the monolayer of NMP (*n*_*m*_ = 18), for which 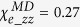, a value more than a hundred times smaller than that for the bulk liquid. The general tendency for 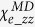 is to slowly increase as the thickness of the confined liquid film is increased. This behaviour is not surprising since the degree of ordering towards the middle point of thicker liquid films is progressively reduced; starting to resemble the bulk liquids. Even if the simulated junctions are too thin to show it, there should exist a thickness above which the results would be indistinguishable from the bulk.

As discussed in the previous section, the layered structure of confined NMP is more complex than that of other liquids. The cell containing 22 NMP molecules included in the simulations at various electric fields is one for which its molecules assume the “standing” position. One may think that in this configuration–where dipoles seem to be already forced into a reduced set of orientations, half of them having a significant component in the direction (+*z*) of the electric field–it would be easier for the electric field to force most of the dipoles to adopt the positive angle and result in increased polarizabilities, perhaps even larger than for the bulk. Table 3 shows, however, that 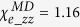 for this case. When compared to the 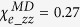 value from the previous configuration (*n*_*m*_ = 18) this is a significant increase, indeed, the largest one in the table. Despite the considerable increase in polarizability seen in the *n*_*m*_ = 22 configuration, its value is still one order of magnitude lower than for the bulk; pointing to the preponderance of confinement-induced order.

The MD work of Martí *et al.*^20^,^21^ reported an increase in water’s dielectric constant when confined between two graphene layers. Their result seems to contradict our findings for the organic polar liquids tested. To test if this discrepancy was due to the nature of the liquids, a set of simulations was run for a monolayer of water confined in our model of an interbundle junction (*n*_*m*_ = 77) and a value of 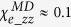 was found. This result, in total disagreement with Martí *et al.*, shows the most significant quenching of polarizability of all the liquids tested. The origin of this discrepancy can be understood by closer inspection of the method of Martí *et al.* In their work they did not apply any electric fields to their simulations; their prediction was based on the average value of the magnitude of the total dipole,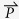, of the confined liquid at zero field. This method implicitly assumes that the medium is isotropic and there are no preferential orientations for 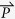 and would fail to predict any anisotropic effects. As shown by our results, the systems under investigation are highly anisotropic and *P*_*z*_ is strongly reduced by confinement. The fact they even found an increase in *ε*_*r*_ may be explained by considering (see SI. 10) that forcing molecules to keep their dipoles confined to a plane may result in larger values of 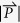, albeit with negligible contributions from *P*_*z*_. The predictions of Martí *et al.* should still hold for electric fields running parallel to the graphene layers, but will clearly fail for fields in the direction normal to the planes.

Table 3 also summarizes the expected contributions of the electronic polarizability to the electric susceptibility in the transverse direction, 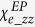. These values, neglected in the non-polarizable MD simulations, were estimated from the polarizability tensors of individual molecules calculated by DFT (see SI. 9.2). Even if 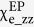 is reduced by confinement, it is not as strongly quenched as 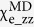; the maximum reduction being ~35% (for NMP with *n*_*m*_ = 18) and not an order of magnitude. Since the static dielectric properties of polar liquids are dominated by the orientational polarizability of their molecules (measured by 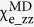) the very subtle changes in 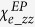 represent only a minor correction, likely to be totally obscured by the massive changes in 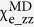.

### Implications for immersed CNT fibres and similar materials

In previous work^7^,^8^,^9^, we experimentally studied the interactions between direct-spun CNT fibres^27^, and organic liquids. We found that liquids with surface tensions ≲50 mN m^−1^ infiltrate the fibres, readily filling the pores in between CNT bundles. The lower specific surface energy between CNT bundles and the liquids (relative to air) triggers a structural relaxation, causing significant changes in the fibres’ electrical conductivities; the final values depending on the total number of open junctions (*i.e.* where the bundles are not in contact) and the ease with which carriers can tunnel through them^7^. Furthermore, fibres immersed in polar liquids were found to exhibit a “non-Ohmic” behaviour in which their conductivity is modulated by an applied electric field^8^,^9^. We attributed this effect to the electrostatic force due to the accumulation of charge at junctions bringing the bundles closer together, thus improving the electrical conductivity.

In general, the presence of a dielectric in a gap between two electrodes (the bundles in a junction in this case) has the effect of reducing the total tunnelling current because the polarization induced in the dielectric opposes the field across the junction, increasing the tunnelling barrier. The confinement-induced reductions in *χ*_*e*_*zz*_ reported in this work are thus expected to result in an increased electronic tunnelling across open interbundle junctions and higher than expected conductivities for fibres immersed in liquids exhibiting this behaviour. On the other hand, the change in *χ*_*e*_*zz*_ will also result in a weakening of the electrostatic forces across open junctions, resulting in a weaker than expected “non-Ohmic” response in immersed fibres. In order to corroborate these predictions, it would be required to test fibres in a pair of (as yet unknown) liquids that, being otherwise identical, exhibit a significantly different reduction of *χ*_*e*_*zz*_ under confinement. It should be noted that effects similar to those described for our direct-spun CNT fibres should occur in similar hierarchically-structured assemblies of carbon nanomaterials such as graphene fibres^28^, twist-spun CNT fibres^29^, cotton-like materials^30^, and buckypapers.

## Discussion

We used modelling techniques to explore the behaviour of several liquids confined to graphene slit pores with widths between 5 Å and 40 Å at room temperature and atmospheric pressure. All liquids were seen to organise into layers parallel to the confining graphitic surfaces, with polar solvents further aligning their electric dipoles parallel to such surfaces. Liquids also exhibited varying degrees of in-plane ordering of the individual layers in the liquid films. In the thinnest films, monolayers of NMP showed remarkable in-plane ordering and an approximately 600-fold reduced molecular mobility, suggesting the existence of a previously unknown 2D solid-like phase with hexagonal symmetry stable under confinement. The tendency of polar liquids to orient their molecular dipoles along preferential directions (mainly parallel to the graphene sheets) resulted in decreases of more than one order of magnitude in their dielectric permittivities (measured as polarizabilities and susceptibilities) in the direction normal to the graphene. This quenching of their dielectric constants should result in improved electron tunnelling across the slit-pores and play an important role determining the electrical conductivity of immersed CNT fibres and other hierarchically-structured nanocarbon-based materials.

## Methods

### Molecular dynamics simulations

All molecular dynamics (MD) simulations reported in this work were run using the “NAnoscale Molecular Dynamics program” (NAMD^31^) version 2.10 and the non-polarizable All-Atom Optimized Potential for Liquid Simulations (OPLS-aa^32^,^33^), an empirical force field designed to reproduce experimental properties of liquids while also matching gas phase torsional profiles. Simulations were run under NpT conditions (at 298 K and 1 bar) and orthorhombic periodic boundary conditions. Temperature was controlled by a Langevin thermostat, while a Langevin piston barostat was used to regulate the pressure. For the non-bonded interactions, switching and cut-off distances of 14.0 Å and 12.0 Å, respectively, were used. When required, static electric fields in the *z*-direction were applied using the built-in functionality of the NAMD code. The time step of each cycle was set to 2 fs and the system was allowed to evolve for 5 million steps (10 ns) capturing a snapshot every 1000 cycles (2 ps). Parameters such as temperature, volume, and enthalpy were generally seen to stabilise after the first 2 ns and analysis was performed on the final 2 ns of simulation time. Prior to simulating the liquids inside the slit pores, reference simulations were run for the bulk liquids, details of which can be found in section 1 of the supporting information (SI. 1).

### Model of the slit pore

Figure 1(a) shows the structure of our slit pore model, which consists of a film of liquid molecules “sandwiched” between two blocks of Bernal-stacked graphite (with 4 graphene sheets in each block). A top-view of a simulation cell, with lateral periodic boundaries marked with red dashed lines, is presented in Fig. 1(b); the stacking of the topmost graphene sheets is clearly visible. Due to the high in-plane rigidity of the graphene, the arrangement of the box contents resulted in an anisotropic response to pressure–virtually all changes in cell dimensions occurred uniaxially in the *z*-direction. The thickness of the liquid film, *c*, calculated by subtracting 26.8 Å (accounting for the 8 graphene sheets) from the total *z*-size of the simulation cell, was allowed to vary in function of the number of molecules in the film, *n*_*m*_. The number and size of the graphene sheets determine the minimum size of our simulation cell and were chosen, having the potential’s cut-off distance in mind, to keep the simulations as small as possible while still preventing interactions of liquid molecules with their periodic self-images.

A schematic of the cross-section of an open junction between two bundles of CNTs is presented in Fig. 1(c). Taking a look at the magnified region (curvature drawn to scale) it can be seen that the narrowest part of the junction appears locally flat at the scale of our simulation cell. Assuming bundles have a circular cross-section it is possible to show (see SI. 2) that the maximum change in junction thickness (from the centre of the junction to either side of the magnified section) is 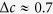 Å, or approximately one-half of the length of a conjugated carbon-carbon bond, which is sufficiently small that not even a single water molecule (the smallest solvent used in this study) would fit in it. Since electron tunnelling is the main transport mechanism across open junctions and tunnelling probability decays exponentially with increasing distance, it is sound to assume that the “locally flat” narrowest section of the junction will have a significant contribution to the overall conductivity. Thus, our model seems reasonable for a basic study of their electrical properties. Furthermore, it will be particularly effective for describing junctions in bundles composed of the collapsed “dog-bone” nanotubes, first observed by Motta *et al.*^34^.

### DFT calculation of molecules’ polarizability tensors

To complement the non-polarizable classical MD simulations, the electronic polarizability tensors of individual molecules were calculated using Gaussian09^35^. The atomic coordinates of the molecules, generated with Materials Studio were passed to Gaussian09 and geometry optimizations, followed by frequency calculations at a temperature of 298 K, were performed. For the calculations, a DFT method with the B3LYP hybrid functional^36^,^37^,^38^, and the cc-pVDZ polarizable double zeta basis set^39^,^40^,^41^,^42^,^43^, were used. By default, frequency calculations return the polarizability tensor and an estimation of the isotropic polarizability of the system as part of the output.

## Additional Information

**How to cite this article**: Terrones, J. *et al.* Enhanced ordering reduces electric susceptibility of liquids confined to graphene slit pores. *Sci. Rep.* **6**, 27406; doi: 10.1038/srep27406 (2016).

## Supplementary Material

Supplementary Information

## Figures and Tables

**Table 1:** Structural parameters of liquid films: interlayer distance (d_il_), in-plane distance to nearest neighbours (d_nn_), effective Lennard Jones diameter (D_LJ_, see^22^).

liquid	*d*_*il*_ [Å]	*d*_*nn*_ [Å]	*D*_*LJ*_^b^ [Å]	*D*_*LJ*_*-d*_*il*_ [Å]	*D*_*LJ*_*-d*_*nn*_ [Å]
CCl_4_	5.1 ± 0.3	4.6 ± 0.2	5.3	0.2 ± 0.3	0.7 ± 0.2
acetone	4.6 ± 0.8	4.8 ± 0.4	4.8	0.2 ± 0.8	0.0 ± 0.4
ethanol	4.0 ± 0.1	3.9 ± 0.2	4.4	0.4 ± 0.1	0.5 ± 0.2
methanol	3.9 ± 0.4	3.4 ± 0.2	3.8	−0.1 ± 0.4	0.4 ± 0.2
NMP^a^	3.8 ± 0.03	6.0 ± 0.3	N/A	≥2.2 ± 0.3	≥0
water	2.5 ± 0.1	2.6 ± 0.2	2.9	0.4 ± 0.1	0.3 ± 0.2

^a^N-methyl-2-pyrrolidone.

^b^Data from^22^.

**Table 2:** Slope of mean square displacement (MSD) in the xy plane vs. time plots for simulated liquids.

liquid	MSD slope [Å^2^ ns^−1^]			bulk/monolayer
	bulk	monolayer	~8 layers	
CCl_4_	413.63	331.55	387.30	1.2
acetone	895.40	30.35	630.45	29.5
ethanol	543.75	34.25	323.32	15.9
methanol	968.79	80.75	789.57	12.0
NMP	63.92	0.10	50.91	634.1
water	1919.92	603.43	1963.08	3.2

**Table 3:** Electric susceptibility in the transverse direction for liquids confined within graphene slit pores.

liquid	c [Å]	n_m_	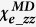		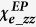	
			value	% of bulk	value	% of bulk
acetone	5.1	25	0.8	5.0	0.38	69
	9.0	47	1.2	7.2	0.43	78
	14.2	75	1.2	7.2	0.49	88
	33.4	187	2.0	12.1	0.53	96
ethanol	5.9	35	1.6	7.4	0.44	82
	13.4	88	1.6	7.4	0.49	92
	33.7	235	1.9	8.7	0.53	98
methanol	5.1	43	1.2	3.6	0.36	81
	8.7	79	1.2	3.6	0.40	90
	13.3	128	1.6	4.9	0.43	97
	32.5	315	1.9	6.0	0.43	97
NMP	5.1	18	0.3	0.9	0.46	65
	7.2	22	1.2	4.1	0.59	82
	34.7	144	1.8	6.1	0.66	93

Super-indices MD and EP indicate results obtained from molecular dynamics and “electronic polarizability” DFT calculations respectively. Columns to the right of the values of indicate their relative sizes (in %) compared to the results for bulk liquids.
