# Supplementary material for: Enhanced ordering reduces electric susceptibility of liquids confined to graphene slit pores
Source: Sci Rep. 2016 Jun 6;6:27406. doi: 10.1038/srep27406 (PMC4893708; doi:10.1038/srep27406)
Supplement: Supplementary Information [file srep27406-s1.docx]

*Supporting information for:*

Enhanced ordering reduces electric susceptibility of liquids confined to graphene slit pores

Jeronimo Terrones, Patrick J. Kiley, and James A. Elliott

Department of Materials Science and Metallurgy, University of Cambridge, 27 Charles Babbage Road, Cambridge CB3 0FS, United Kingdom.

# Simulations of bulk liquids

In this case, a box being 26×26 Å in the base (*xy*-plane) and 30 Å in height (*z*-direction) was filled with a number of molecules consistent with the expected density of the liquid to be tested. Figure S1 shows the cell used methanol, the periodic box is marked in blue. The cell dimensions were selected to match those of the simulated slit pores.

#
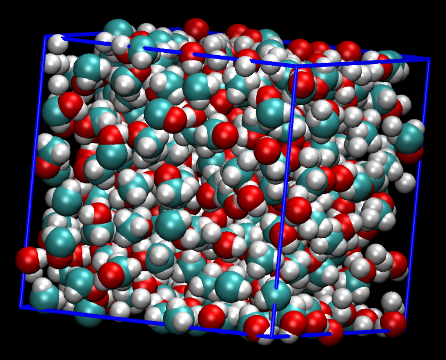


Figure S1. Initial simulation conditions for bulk methanol; the periodic box is marked in blue.

Test simulations were then run with the same parameters used for simulating the slit pores, also allowing the *z*-dimension of the cell to change to adjust the pressure. Table 1 compares the density values calculated from the last 2 ns of each simulation with values from literature ^1^ for the different liquids. The agreement seems very reasonable for all liquids, with the maximum discrepancy being −3.3% for methanol. The model seems to work remarkably well with ethanol and shows only a 0.02% deviation; a difference completely within the statistical error of the simulation’s prediction.

Table 1. Comparison between the mass densities calculated from the MD simulations and experimentally determined values from literature ^1^.

| **Liquid** | **Calculated Density [g cm^−3^]** | **+/- [g** **cm^−3^]** | **Exp. Density [g cm^−3^]** | **Difference [%]** |
| --- | --- | --- | --- | --- |
| CCl_4_ | 1.549 | 0.02 | 1.587 | −2.35 |
| Acetone | 0.803 | 0.01 | 0.791 | 1.52 |
| Ethanol | 0.789 | 0.01 | 0.789 | 0.02 |
| Methanol | 0.766 | 0.01 | 0.792 | −3.28 |
| NMP | 1.015 | 0.01 | 1.028 | −1.24 |
| Water | 0.986 | 0.01 | 1.000 | −1.41 |

# Calculation of *Δc*

Figure S2 shows a schematic of the cross section of two round bundles of diameter $D_{b}$ (represented as circles): the sizes of *b* and *c* are exaggerated to make the discussion clearer. From the schematic it can easily be seen that:

|  | $\Delta c=D_{b}-2K$. | (S1) |
| --- | --- | --- |

$K$ can be calculated from the triangle with sides $K$, $b/2$, and $D_{b}/2$ as:

|  | $\Delta c=\sqrt{\left( \frac{D_{b}}{2} \right)^{2}-\left( \frac{b}{2} \right)^{2}}$. | (S2) |
| --- | --- | --- |

Substituting (S2) in (S1) and simplifying results in:

|  | $\Delta c=D_{b}-\sqrt{D_{b}^{2}-b^{2}}$. | (S3) |
| --- | --- | --- |

This means that, for $D_{b}=300$ Å and $b=20$ Å (a common diameter for CNT bundles ^2^, and the scale of simulation cells, respectively), $\Delta c\approx0.7$ Å, a gap so thin that not even a single water molecule would fit in it. This justifies our approximation of planar slit geometry for simulation studies.


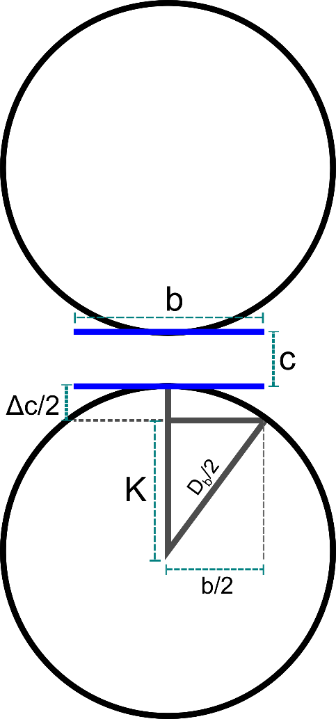


Figure S2. Diagram used to calculate Δc.

# Transverse solvent ordering

## Changes in *ρ*, *c*, and *H* as a function of *n_m_.*

Figure S3 shows how density (*ρ)*, film thickness (*c*), and enthalpy (*H*) change in (a) carbon tetrachloride, (b) acetone, and (c) ethanol as the number of liquid molecules ($n_{m}$) is increased; Figure S4 does the same for (a) methanol, (b) NMP, and (c) water. The density and enthalpy values obtained from the simulations of bulk liquids, along with their statistical uncertainties, are indicated with dashed lines on each plot. It can be seen that, in all cases, densities and enthalpies tend to the values for the bulk as the thickness of the film increases.

## Additional *z*-distribution profiles

The histograms in Figure S5 show the distribution of the geometric centres of the molecules (measured with VMD, ^3^) along the *z*-direction (*i.e.* the transverse direction) for increasingly thicker films of (a) carbon tetrachloride, (b) acetone, (c) ethanol, (d) methanol, and (e) water. In all cases, the sharpest peaks, indicating the most ordered layers, are those in contact with the graphene surfaces. The slightly more complicated behaviour of *N*-methyl-2-pyrrolidone (NMP) is explored in detail in Figure S6. For all cases, order is gradually lost towards the centre, especially in the case of thicker films. The degree of ordering and the rate at which it decays with distance from the graphene seems to depend on the particular liquid being simulated.

It should be noted that in each of these figures there is a mismatch between the given value of the thickness of the liquid film, *c*, and the thickness that can be measured from the spread of the histogram. This occurs because the *z*-size of the histograms is that between the bottommost molecular centre (set to be at 0) and the topmost one, whereas the measurements of *c* include the whole volume occupied by the molecules (giving an additional thickness of ~4 Å).


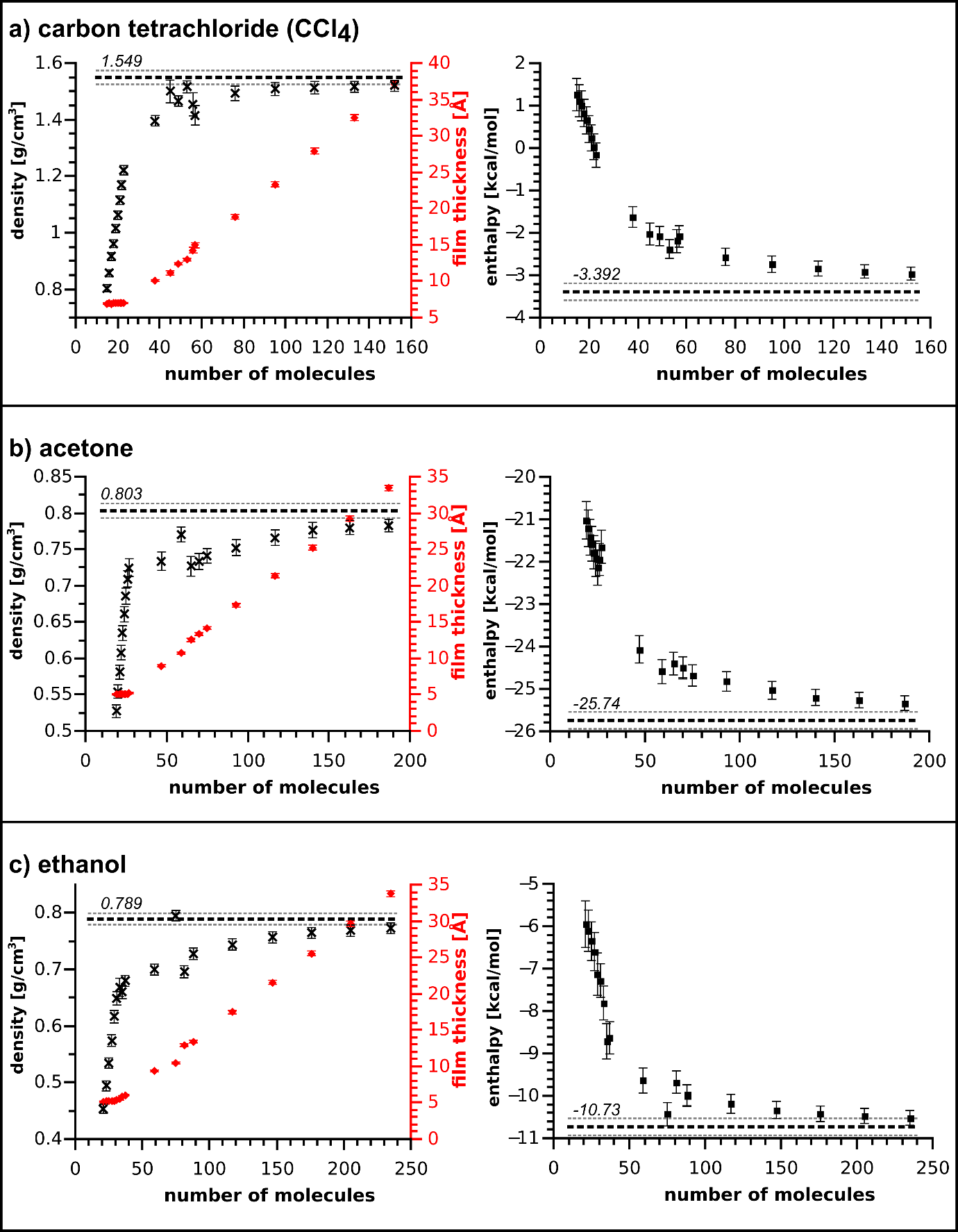


Figure S3. Changes in the liquid density (left plot, black data), thickness of the liquid film (left plot, red data), and enthalpy (right plot) as a function of the number of molecules in the film. Data for (a) carbon tetrachloride, (b) acetone, and (c) ethanol.


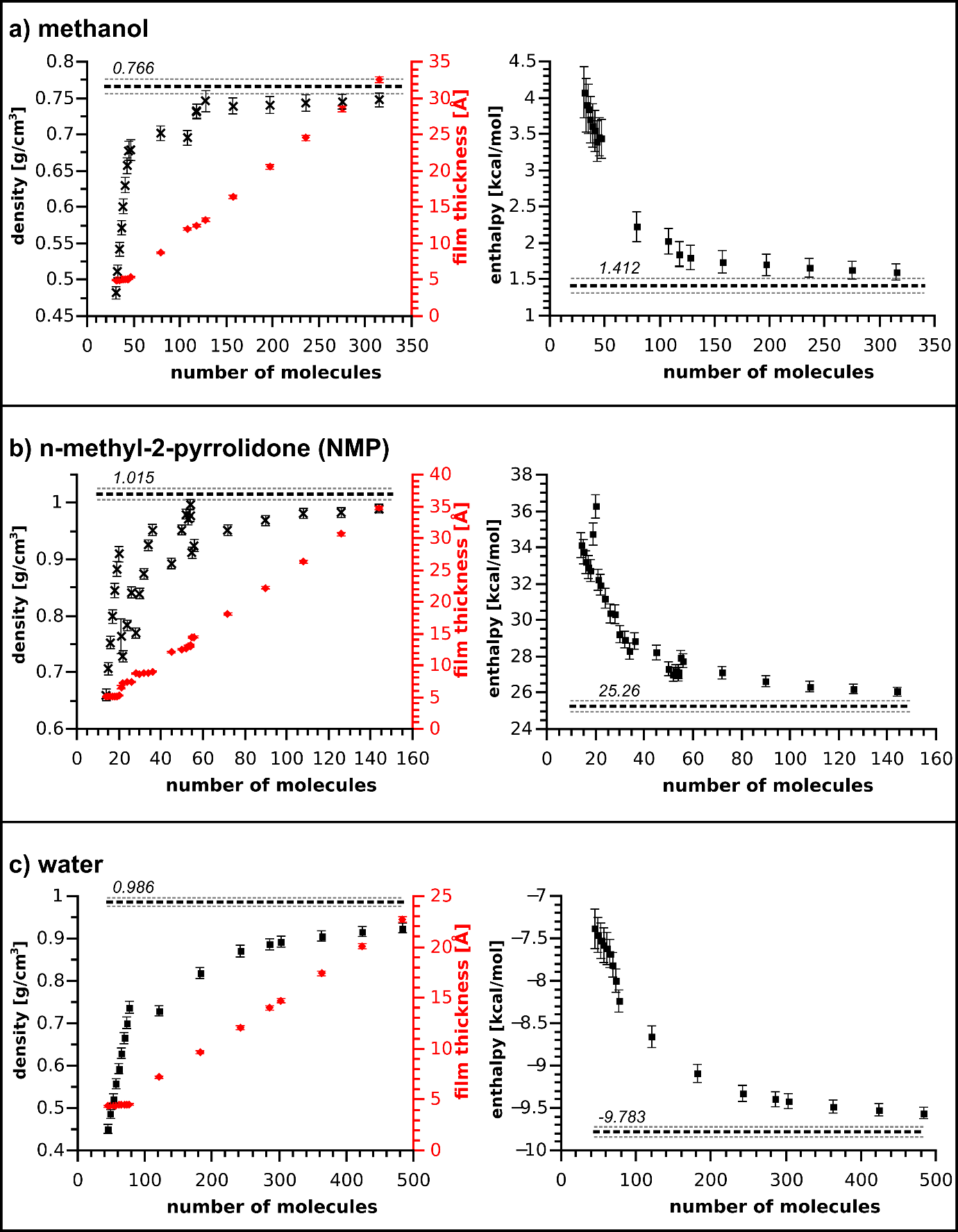


Figure S4. Changes in the liquid density (left plot, black data), thickness of the liquid film (left plot, red data), and enthalpy (right plot) as a function of the number of molecules in the film. Data for (a) methanol, (b) N-methyl-2-pyrrolidone, and (c) water.


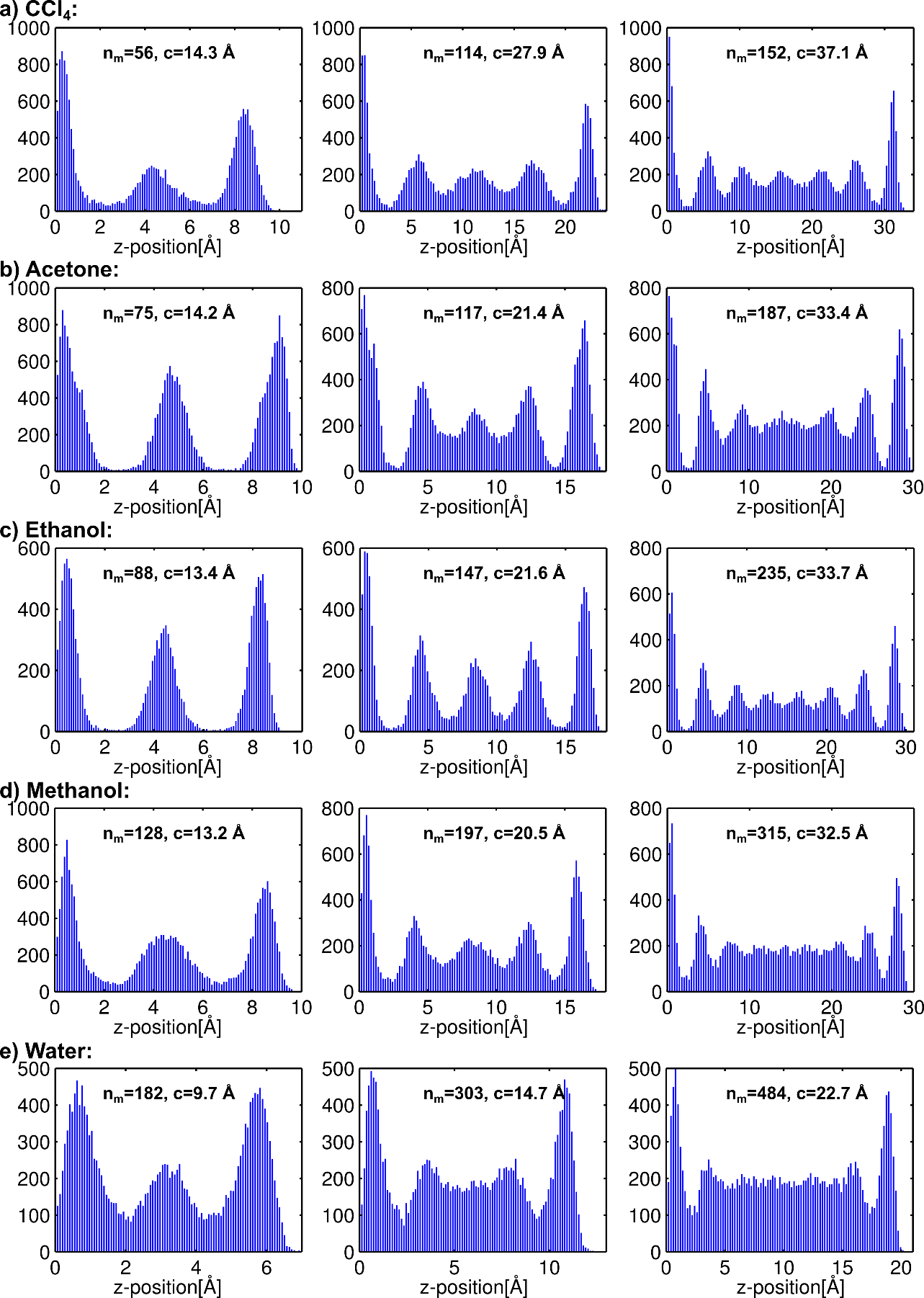


Figure S5. Distribution of the geometric centres of liquid molecules across increasingly thicker (increasing $n_{m}$) interbundle films for: (a) carbon tetrachloride, (b) acetone, (c) ethanol, (d) methanol, and (e) water.


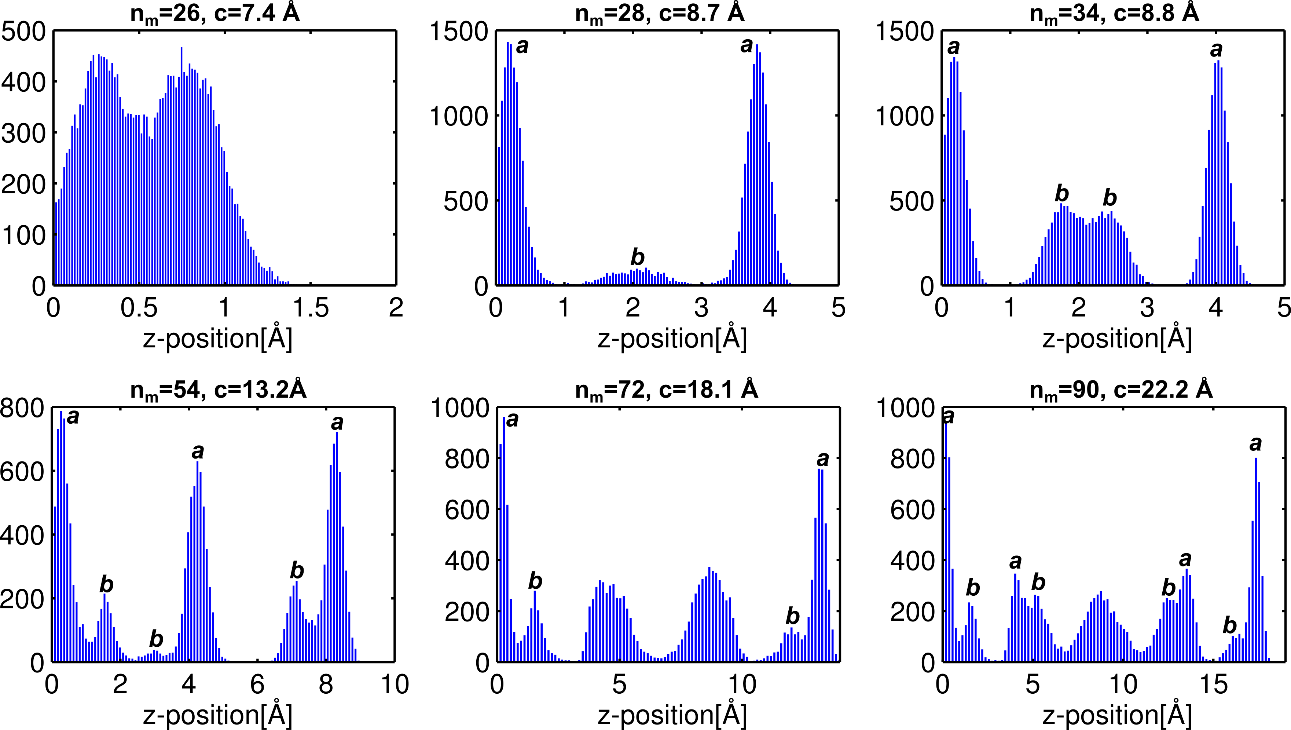


Figure S6. Distribution of the geometric centres of NMP molecules across increasingly thicker films; notice the appearance of distinct peaks a and b and the plane of symmetry at the centre of the liquid film.

# Additional pair distribution functions (PDFs)

Figure S7 shows *xy* pair distribution functions (PDF) for the geometric centres of liquid molecules in the thinnest films, where only a molecule-thin layer of liquid is separates the graphene sheets; in this figure, the darkest areas represent the zones of highest probability to find a molecule.


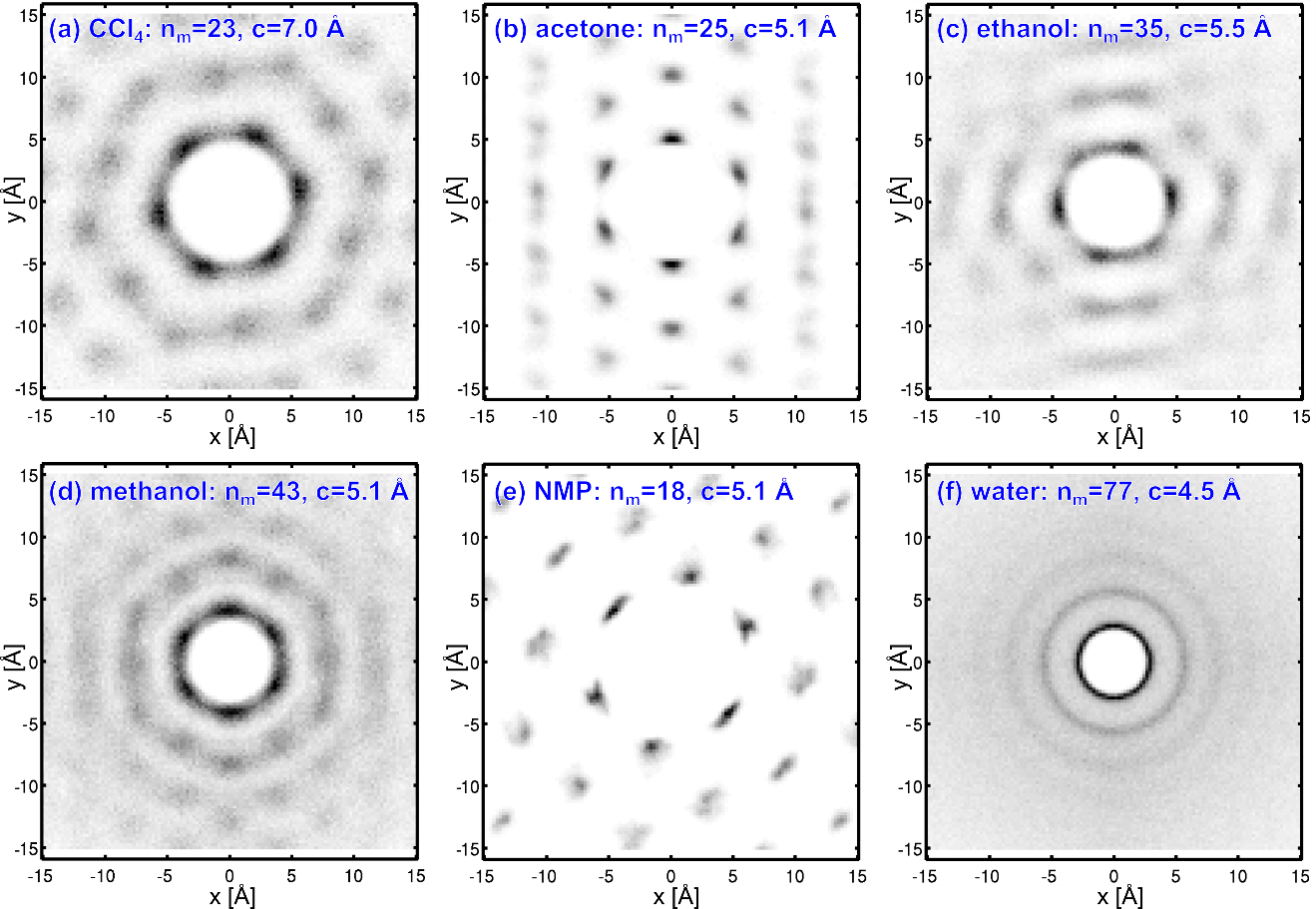


Figure S7. Distribution of the geometric centres of solvent molecules in the x-y plane.

Figure S8 shows PDFs of individual layers within the thickest films of (a) acetone, (b) NMP, and (c) water. The top (1) and bottom (3) rows of panels in the figure correspond respectively to the topmost and bottommost layers of molecules (both in contact with the graphene sheets) whereas the middle row (2) corresponds to layers towards the centre of the film. Two things can be noted from this picture: ordering in the layers in contact with graphene is not as good as in monolayers (compare with Figure S7) and the order further decreases towards the centre of the films. Similar behaviours were observed in the other three liquids.


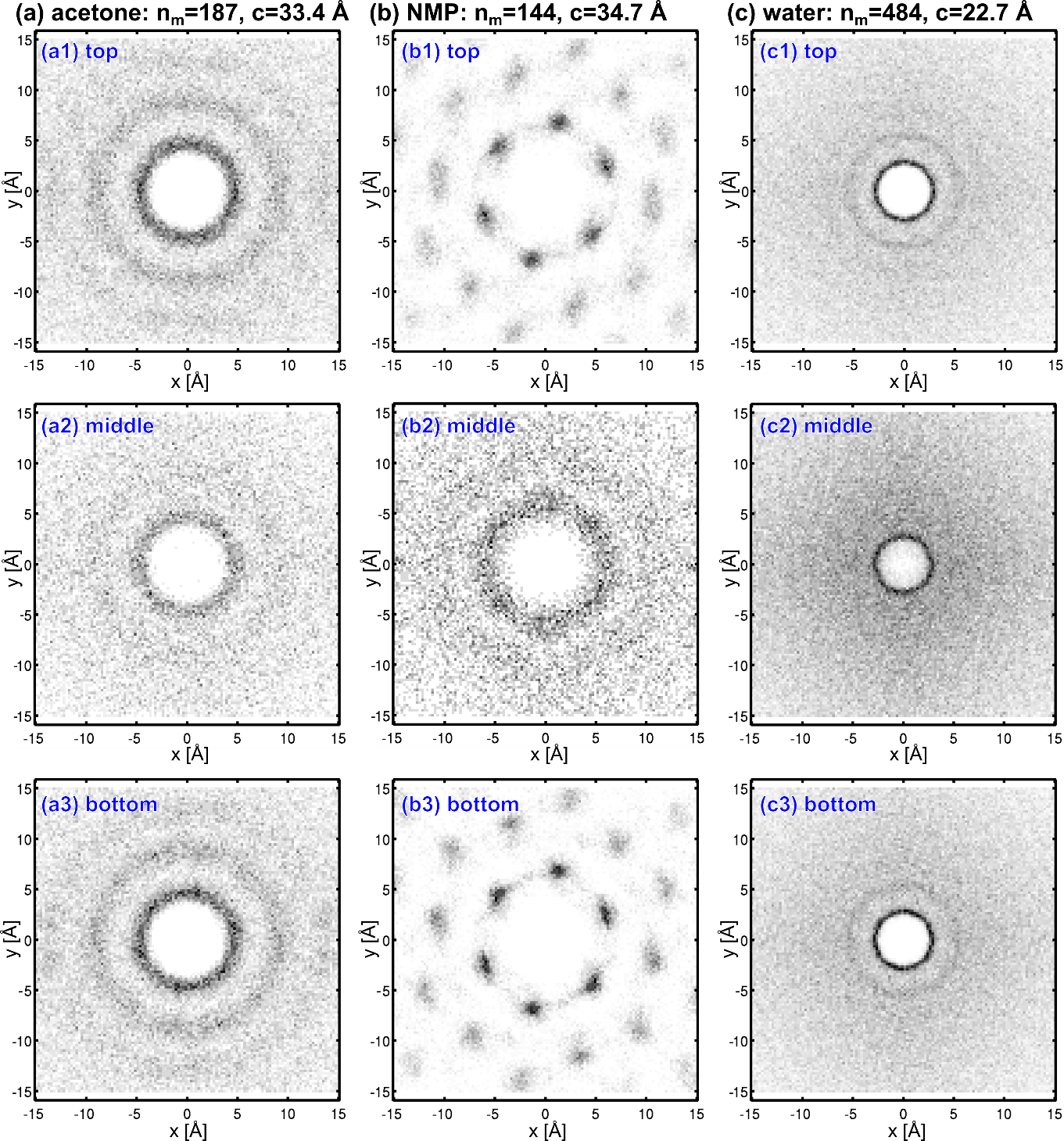


Figure S8. xy-PDFs of individual layers within the thickest films of (a) acetone, (b) NMP, and (c) water. The top (1) and bottom (3) rows of panels in the figure correspond respectively to the topmost and bottommost layers of molecules (both in contact with the graphene sheets) whereas the middle row (2) corresponds to layers towards the centre of the film.

Figure S9 explores how the positions of molecules in a single layer correlate to those in the contiguous layer for the case of double-layer films. These modified PDFs show the relative positions of molecules in the bottom layer with themselves (cyan) and the molecules of the top layer (magenta). For all molecules but water there is a tendency of molecules in one layer to avoid being exactly on top of a molecule in the other layer. This tendency is exceptionally marked in CCl_4_ (with very-well defined pure magenta and cyan spots) and seems to be progressively lost as the size of the molecule decreases (Table 1 in the main text can be used as a reference); being completely lost for water, where the distribution of magenta seems completely random – note that even if NMP shows a larger diameter than CCl_4_ it is a thinner molecule so it may be considered slightly smaller (at least in the *z*-direction).


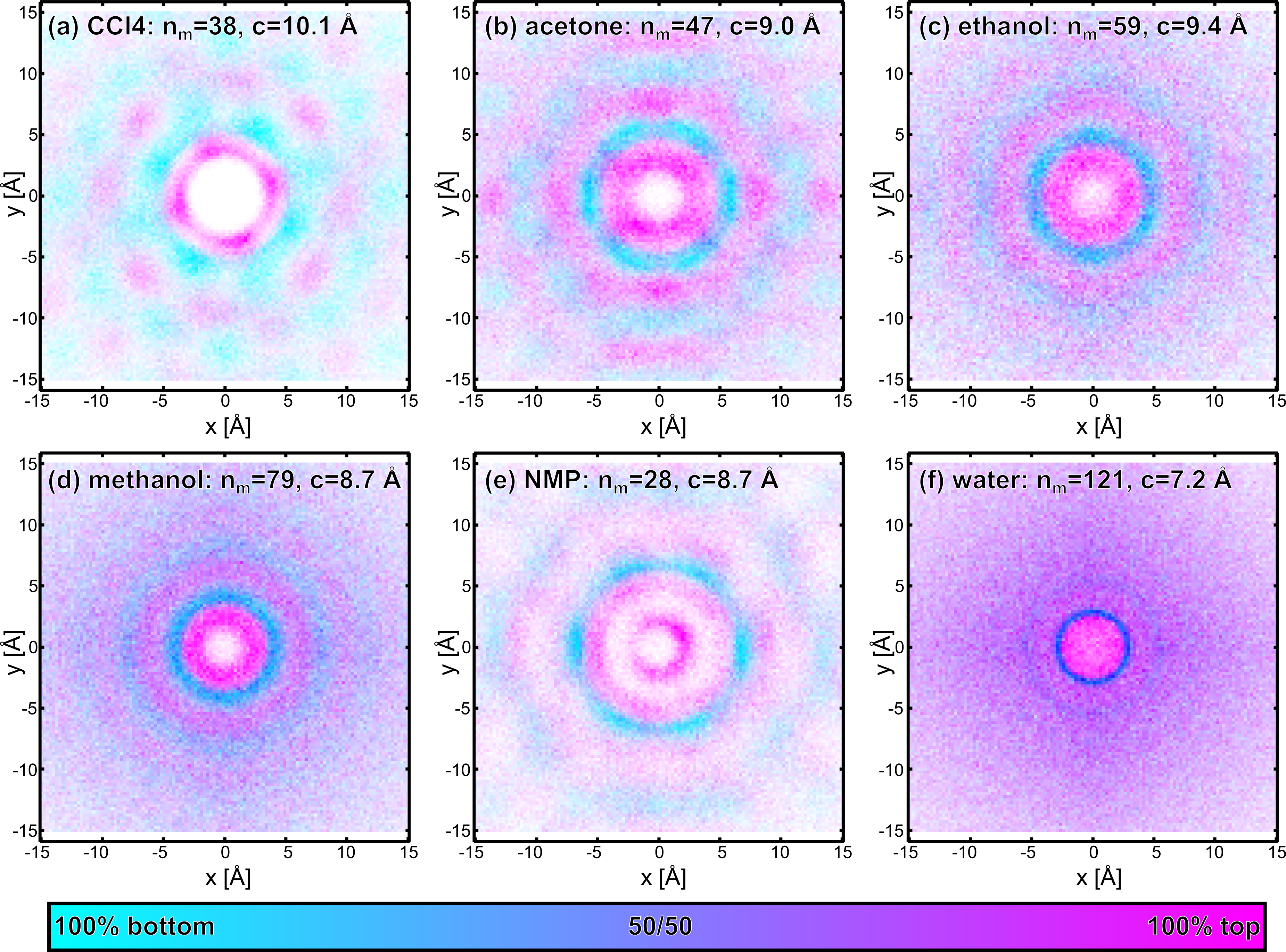


Figure S9. Distribution of molecules for bi-layer cases. PDFs are calculated with respect to molecules in the bottom layer. Molecules in the bottom layer are coloured cyan; molecules in the top layer, magenta.

# Structure of thinnest NMP and acetone monolayers

Figure 3(f) (main text) and Figure S10 show snapshots of the simulations of the thinnest films of NMP and acetone respectively. For convenience all the graphene layers but one (the one immediately below, indicated by grey spheres) have been removed; the green spheres indicate the geometric centres of the molecules. The figures display a great degree of ordering, as would be expected for solid-like or liquid crystalline phases: hexagonal patterns are clearly evident; furthermore, it can be seen that molecules tend to lay flat (as suggested in the case of NMP by the comparison of *d_nn_* to *d_il_*, Table 1 in the main text) and appear to assume specific orientations.


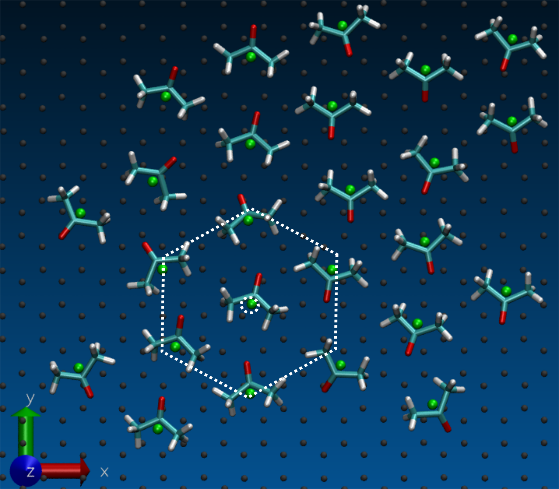


Figure S10. Geometric patterns in the x-y distribution of acetone molecules in the monolayer. Grey spheres indicate the C atoms in the lower graphene sheet. The geometric centres of molecules are marked by green spheres. Notice molecules laying with their long axes parallel to the graphene surface, as suggested by Israelachvili et al. for OMCTS, ^4^, and Shih et al., ^5^, for NMP and other solvents.

# Orientation of polar liquids’ molecular dipoles

## Orientation of dipoles within the molecules

The molecular structures of the six liquids are shown in Figure S11 along with information about their electric dipoles.


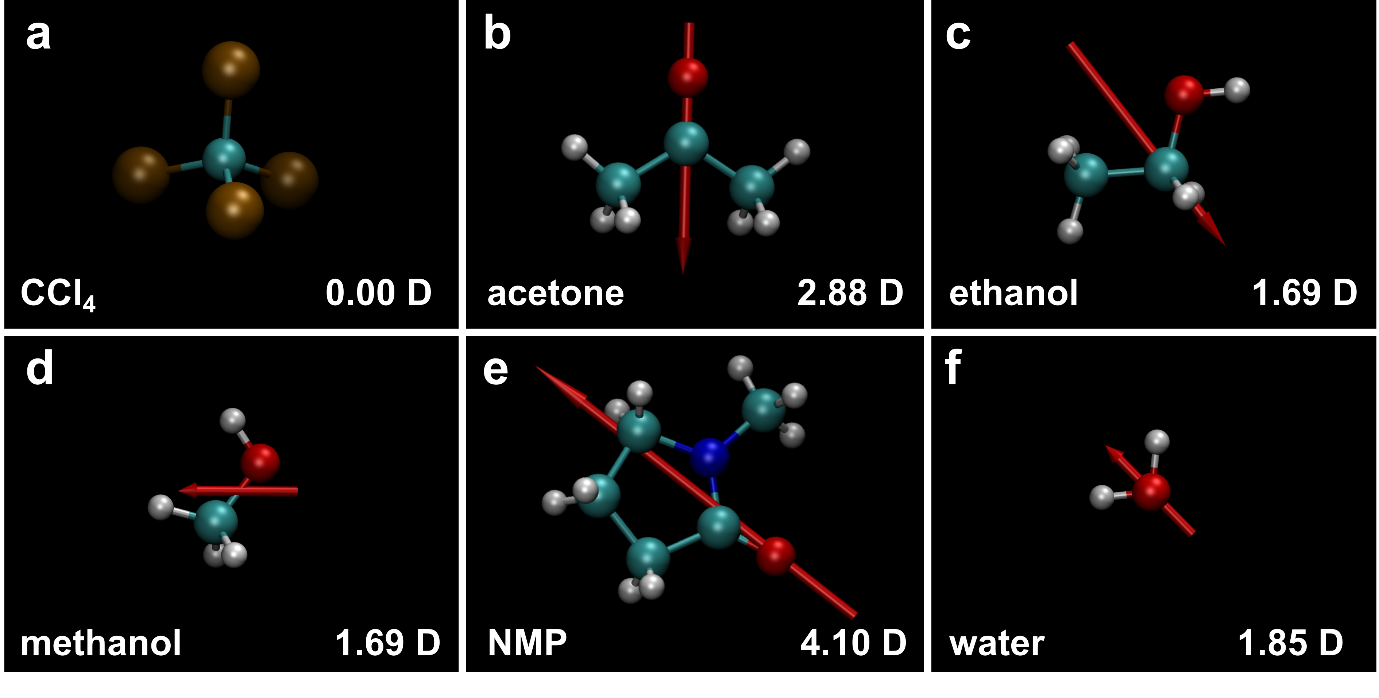


Figure S11. Molecules of liquids used in the simulations: (a) carbon tetrachloride, (b) acetone, (c) ethanol, (d) methanol, (e) N-methyl-pyrrolidone (NMP), and (f) water. The red arrows indicate the direction of the molecules’ electric dipole moments as determined with VMD ^3^; the magnitude of each of the dipoles ^1^ is displayed on the lower right corner of each frame. Colour code: (blue) nitrogen, (cyan) carbon, (bronze) chlorine, (red) oxygen, (white) hydrogen.

## Preparing stereographic plots

VMD was used to extract the dipole data from the simulation trajectories. For convenience, extracted values were then normalized so that the end-points of all dipoles would fall in the surface of a unit sphere. For display purposes, a stereographic projection was then used to map the unit sphere to the plane. The mapping function used is:

|  | $\left( x',y' \right)=\left( \frac{x}{1+\left\vert z \right\vert},\frac{y}{1+\left\vert z \right\vert} \right)$ | (S4) |
| --- | --- | --- |

where $\left( x,y,z \right)$ are the coordinates of the vectors and $\left( x',y' \right)$ are the projected coordinates. This formula corresponds to a projection onto the unit circle of the north hemisphere of the unit sphere with respect to the south pole and the south hemisphere with respect to the north pole. Blue and red dots are used in the plots to distinguish between vectors with negative and positive *z* components respectively (the sign of the *z* component defines the sign of the elevation angle, $\varphi$).

## Additional stereographic plots

Figure S12 compares the stereographic plots of the orientation of the liquids’ molecular dipoles in the bulk to those in the thinnest liquid films; it is clear that, while only acetone (a) and NMP (d) show strong azimuthal ordering, all confined liquids orient their dipoles at low elevation angles.

## z-component of the molecular electric dipoles, $\boldsymbol{\mu}_{\boldsymbol{z}}$, for bulk and confined solvents.

Figure S13 shows the distribution of the molecules’ $\mu_{z}/\left| \mu\right|$ for a select number of simulations. Solvents are organised by rows, and the columns (from left to right) correspond to bulk, monolayer, and triple layer cases. From these plots we can observe that the general trend is for dipoles to remain parallel to the *xy*-plane ($\mu_{z}/\left| \mu\right|$ centred around 0), especially for monolayers. As films get thicker, disorder increases and a superposition of the bulk distribution and the monolayer distribution is seen. Ordering is much stronger in NMP and, even in the thickest films (see Figure S14), shows a clear superposition of an ordered peak (due to the layers closest to the graphene surfaces) and a bulk flat background. Furthermore, for the tri-layer configuration ($n_{m}=54$, Figure S13(d)), dipoles in NMP suggest a secondary pair of preferred elevation angles close to ±50˚, these peaks correspond to the “standing” orientation responsible for the “subsidiary” *B* peaks in the *z*-distribution profiles (Figure 2(c) (main text), and Figure S6) .


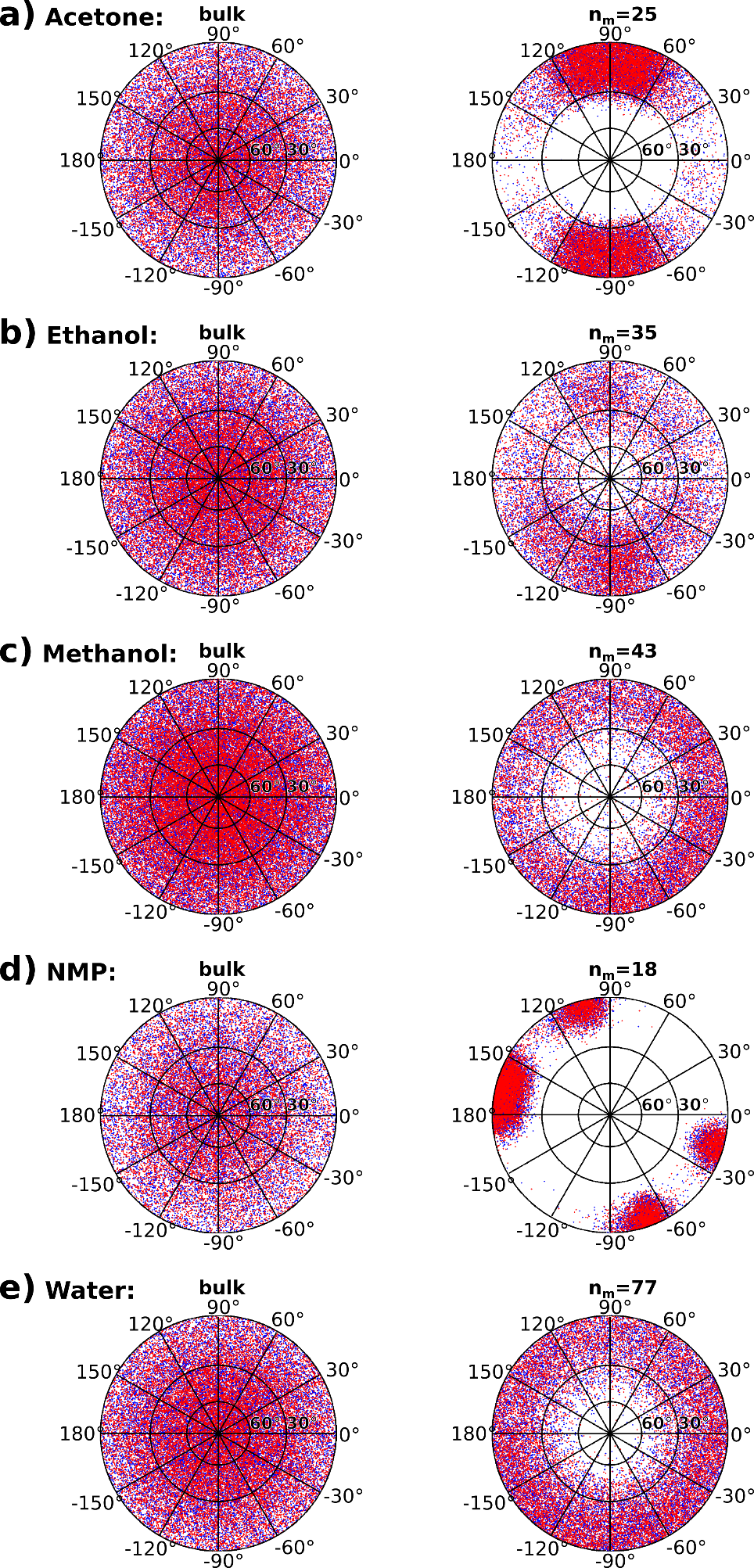


Figure S12. Stereographic plots showing the orientation of the solvent molecules’ electric dipoles in in the bulk and the thinnest films.


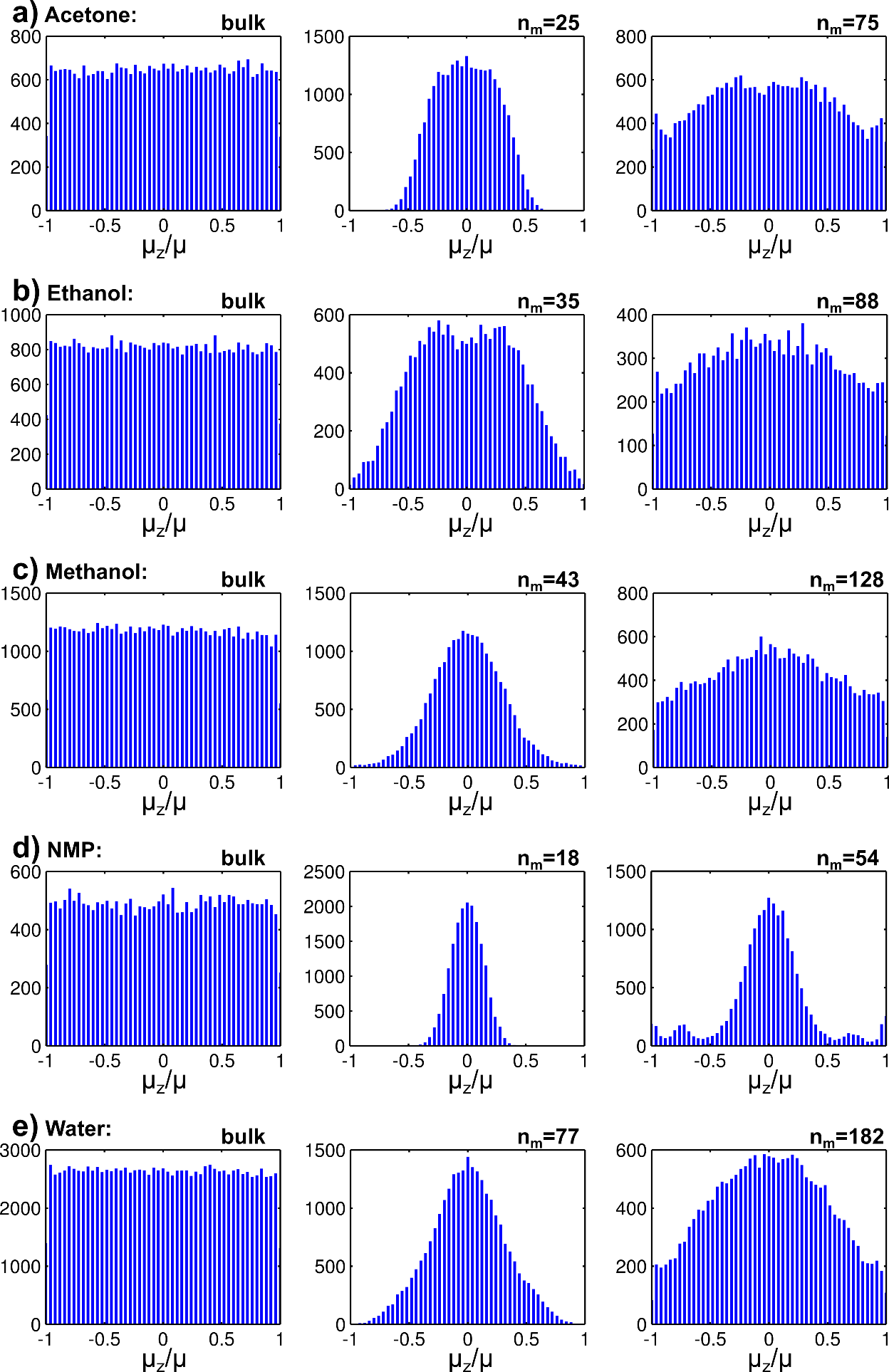


Figure S13. Distribution of the z-component of the molecular electric dipoles for bulk and confined solvents.


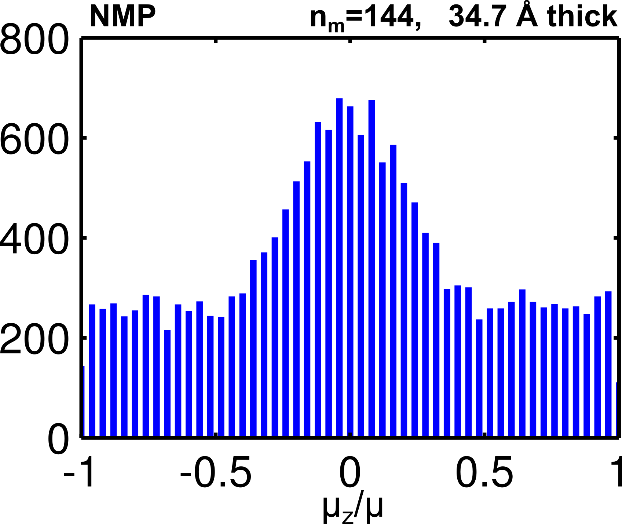


Figure S14. Distribution of the z-component of the electric dipole of NMP molecules in the thickest film simulated.

# A closer look at orientation of NMP molecules

Figure S15 shows – by means of (from left to right) stereographic plots, cell snapshots, and z-distribution plots – the evolution of NMP as the film thickness increases from 5.1 Å (a monolayer) to ~8.7 Å (a bilayer, which has “subsidiary” *B* peaks starting to form). When going from film thicknesses 5.1 Å to 7.4 Å ($n_{m}$ from 18 to 26, Figure S15 (a-c)) we see that the monolayer is preserved, however a shift in orientation occurs. For the thinnest monolayers, molecules will lay flat, with their dipoles parallel to the graphene sheets. As the film thickness increases (indirectly controlled by increasing $n_{m}$), molecules in the monolayer opt for the “standing” orientation, in which the dipoles take elevation angles of ~±50˚. The double peak clearly visible in the *z*-histogram of Figure S15(c) is due to the slightly different position of the geometric centres of molecules with positive and negative elevation angles. As the solvent film’s thickness progressively increases we see the formation of two distinct monolayers of flat-laying molecules (Figure S15(d)) and then two monolayers with the majority of the molecules laying flat but some of them standing (Figure S15(e)). The central peak observed in the *z*-histogram of Figure S15(e) is due to the molecules which dipoles assume elevation angles of ~±50˚. As the solvent film increases further, yet before a third layer of flat NMP molecules is formed, this central peak will split in two, due to some of the “standing” molecules remaining in the top layer and others in the bottom one. This behaviour is the cause of the mirror-symmetric (mirror plane in the centre of the film) subsidiary *B* peaks observed in Figure 2(c) (main text) and Figure S6.


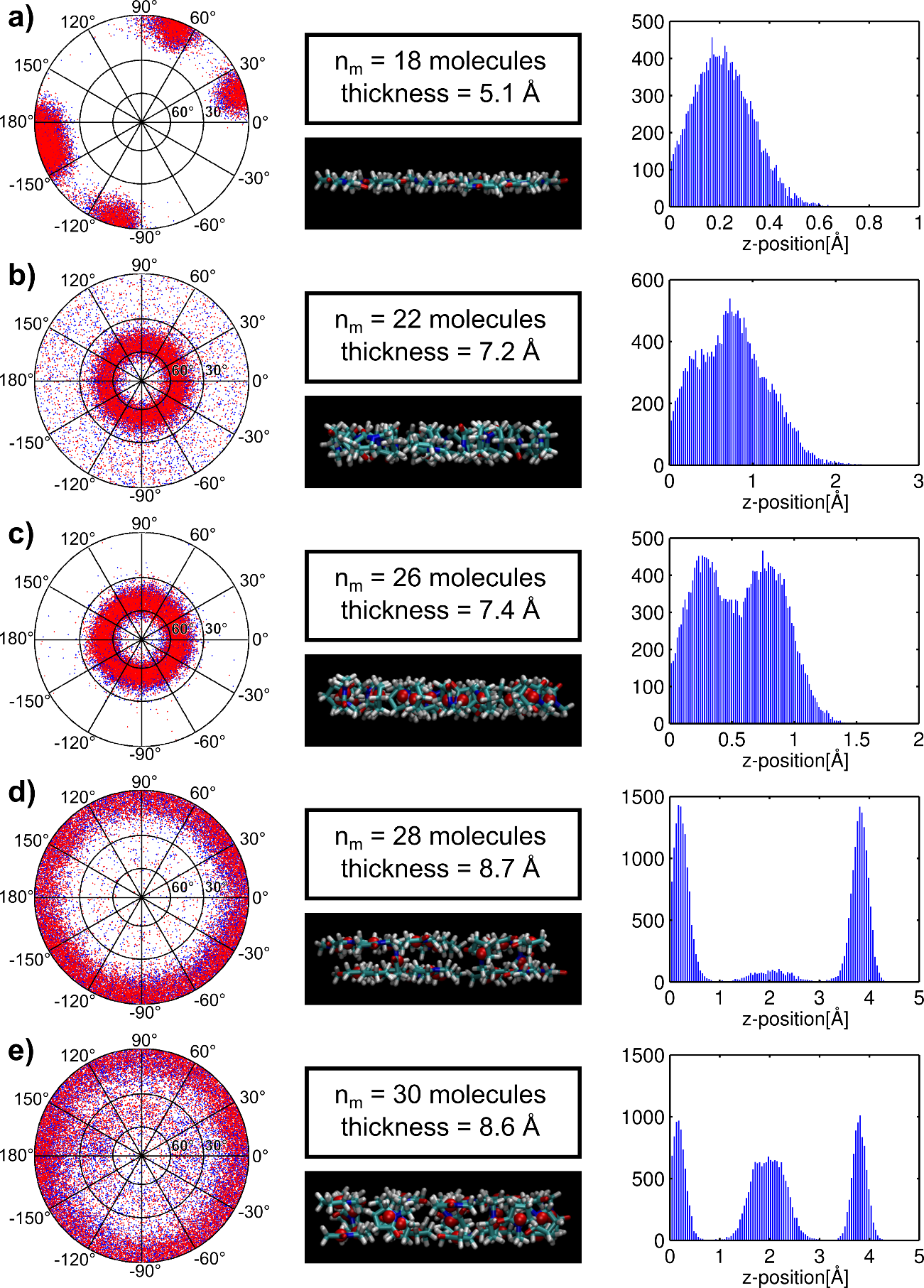


Figure S15. Structure of increasingly thicker films of NMP. Includes: Stereographic plots (left), Snapshot of the simulation (centre), Distribution of molecules in the z-direction (right). For panels c to e, the red spheres indicate the geometric centres of the molecules.

# NMP monolayer with modified aspect ratio

In the bulk, NMP solidifies at -24 ˚C (249 K); its solid form (studied at 168 K, ^6^) has a monoclinic crystal structure with lattice parameters a=6.221 Å, b=12.076 Å, c=7.529 Å, and β=111.03˚, and four molecules in each unit cell. Figure S16 shows a slice of a NMP crystal. It can be seen that he arrangement of molecules differs from that of a confined monolayer (Figure 3(f), main text). As discussed in the main text, this discrepancy could suggest the existence of a different highly-ordered phase of monolayer NMP stable at room temperature under confinement, similar to the case of “square ice”, ^7^. However it is also possible that the aspect ratio and areal densities artificially imposed by the periodic boundary conditions into the simulation cell are favouring a different structure. The slice of the monoclinic crystal of Figure S16 is 24.9×24.2 Å, has an aspect ratio (*x/y*) of 1.03 and contains 16 molecules (*i.e.* 0.027 molecules Å^−2^). The slightly larger, 27×25.5 Å, standard cell of our simulations (see Figure 1(b), main text) has an aspect ratio (*x/y*) of 1.06 and, for the case of a monolayer ($n_{m}=18$), contains 2 extra molecules (*i.e.* 0.026 molecules Å^−2^). The parameters of the two structures are not too different, however, to check the effect of a changing geometry, a new cell was constructed by removing one column of hexagons from each graphene sheet in the standard cell. This test cell was 24.6×25.5 Å, had an aspect ratio (*y/x*, in this case) of 1.04, and (when $n_{m}=16$), an areal density of 0.026; this is the closest that one can get to the geometry of the NMP slice of Figure S16, without having to significantly modify the size of the simulation cell. The PDF and a cell snapshot of the resulting structure are shown in Figure S17. It can be seen that confined NMP molecules still seem to prefer (albeit in another orientation this time) a more hexagonal pattern. This preference further supports the idea of the existence of a stable phase of “hexagonal” NMP in confined systems at room temperature. A full study of the effects of the boundary conditions of the simulation on the structure of NMP is beyond the scope of this paper.


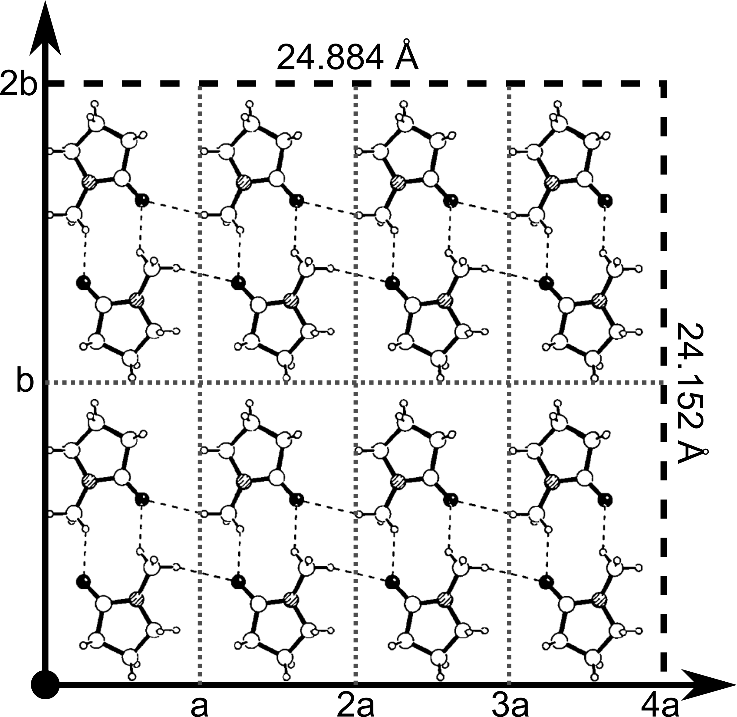


Figure S16. Structure of an NMP crystal at 168 K. Modified from ^6^.


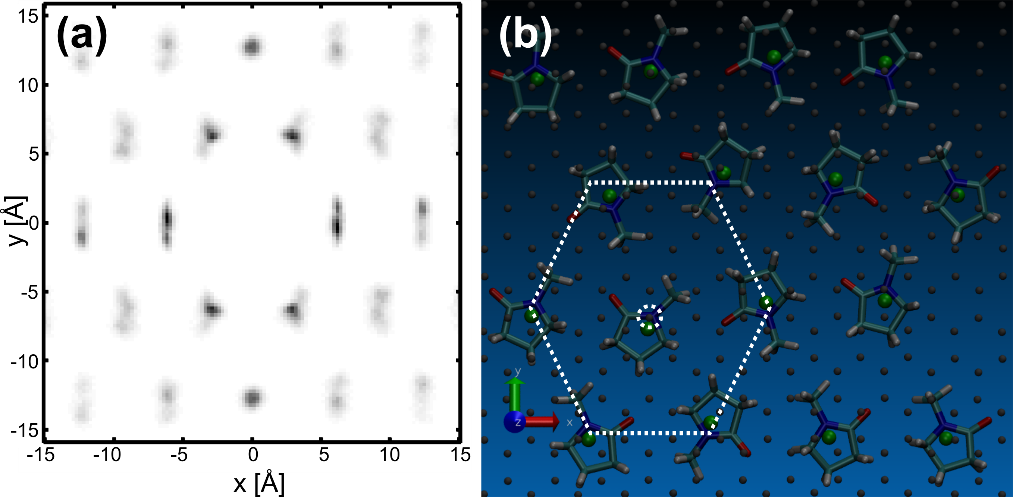


Figure S17. Distribution of 16 NMP molecules in a cell modified to have an x-y aspect ratio as close as possible to that of bulk crystallized NMP. Grey spheres indicate the C atoms in the lower graphene sheet. The geometric centres of molecules are marked by green spheres.

# Determination of the electric susceptibility, $\chi_{e}$

## Calculation of $\boldsymbol{\chi}_{\boldsymbol{e\_zz}}^{\boldsymbol{MD}}$ from the slope of $\boldsymbol{P}_{\boldsymbol{z}}\boldsymbol{/}\boldsymbol{n}_{\boldsymbol{m}}$ *vs.* $\boldsymbol{E}_{\boldsymbol{z}}$ plots

The dipole, $p$, induced by an electric field, $E$, on an individual molecule is given by

|  | $p=\alpha E$ | (S5) |
| --- | --- | --- |

where $\alpha$ is the polarizability of the molecule. To avoid any confusion, it is important to mark the difference between $\mu$ and $p$: while $\mu$ is the permanent dipole of a molecule, originated from the average spatial distribution of charge between its component atoms, $p$ is the dipole induced in the direction of an external electric field. For a group of $n_{m}$ molecules, the total induced dipole is given by:

|  | $P_{induced}=\sum_{i=1}^{n_{m}} p_{i}=\sum_{i=1}^{n_{m}} {\alpha_{i}E}_{i}$ | (S6) |
| --- | --- | --- |

where the electric field in the vicinity of each molecule is allowed to change to account for the “screening” effect of the surrounding molecules. For a bulk liquid however, where molecules of the same species are randomly arranged and constantly changing position, a scalar average isotropic polarizability per molecule, $\bar{\alpha}$, can be defined such that:

|  | $P_{induced}=n_{m}\bar{\alpha}E$ | (S7) |
| --- | --- | --- |

where it is further being assumed that the electric field remains constant through the whole liquid – this is a reasonable assumption for our simulations considering that we are mainly dealing with thin films and even the ‘low’ fields, although below the dielectric strength of water, are considerably strong (of the order of 1 MV m^−1^) to be significantly affected by the screening effect. Under the previous assumption, $\bar{\alpha}$ can be used to estimate the electric susceptibility, $\chi_{e}$, from:

|  | $\chi_{e}=\frac{N\bar{\alpha}}{\varepsilon_{0}}$ | (S8) |
| --- | --- | --- |

where $N$ is the number density of the liquid and $\varepsilon_{0}$ is the permittivity of free space. From equation S7, it should be evident that the slopes of the line fits of Figure 5 (main text) correspond to the values of $\bar{\alpha}$ for their respective liquids. And equation (1), a modified version of S8, can be used to calculate $\chi_{e\_zz}^{MD}$.

## Calculation of $\boldsymbol{\chi}_{\boldsymbol{e\_zz}}^{\boldsymbol{EP}}$

First, a DFT calculation (see “Methods” in main text) was performed in Gaussian09 to obtain the electronic polarizability tensor, $\alpha^{EP}$, of individual liquid molecules in a “standard” orientation – the super-index *EP* will be used for quantities dealing with the effects of electronic polarizability, ignoring re-orientation of molecules’ permanent $\mu$. Then the rotation matrices, $R$, required to re-orient molecules in the saved MD trajectories to Gaussian’s “standard” orientation were calculated with VMD ^3^. Finally, the average effective electronic polarizability tensor (projected into the coordinate system of the MD simulations), $\bar{\alpha}^{EP}$, for each chosen case was obtained from

|  | $\bar{\alpha}^{EP}=\frac{1}{n_{f}n_{m}}\sum_{i=1}^{n_{f}} \sum_{j=1}^{n_{m}} R_{ij}\alpha^{EP}R_{ij}^{-1}$ | (S9) |
| --- | --- | --- |

where $n_{m}$ and $n_{f}$ are respectively the number of solvent molecules being simulated and the number of frames in the MD trajectory over which the average was being calculated (1000 frames for the last 2 ns of each simulation). Please note that the subindices in $R_{ij}$ denote the particular rotation matrix for the j^th^ molecule during the i^th^ time step of the MD simulation and not the individual ($R_{xx}$, $R_{xy}$, $R_{xz}$, etc.) matrix-elements of $R$. The approximation given by equation S9 ignores the specific position of molecules in the simulation cell (otherwise, additional translation matrices would have been required) and the effect of intermolecular interactions on their electronic clouds; however it should provide useful, at least at a qualitative level, information under our assumption of thin liquid films under uniform fields strong enough to overwhelm the screening effects of surrounding molecules. Furthermore, since the orientation of individual molecules didn’t appear to be strongly affected by electric fields below the dielectric strength of water, especially in confined systems, estimations of $\bar{\alpha}^{EP}$ at zero field should still hold reasonably well in the “low-field” regime. Then, equation S8 can be used to calculate $\chi_{e}^{EP}$. The values of $\chi_{e\_zz}^{EP}$ appearing in Table 3 (main text) are those corresponding to the diagonal *z*-component of $\chi_{e}^{EP}$calculated at zero electric field for each of the liquid films listed in the table.

# Increased magnitude of total dipole in confined monolayers

Figure S18 shows histograms of the spherical components (in columns from left to right, azimuthal ($P_{\theta}$), elevation ($P_{\varphi}$), and radial ($P_{r}$)) of the total dipole per molecule

|  | $P=\frac{1}{n_{m}}\sum_{i=1}^{n_{m}} \mu_{i}$ | (S10) |
| --- | --- | --- |

in simulations of bulk (a) and the thinnest monolayer (b) of NMP at zero electric field. In this case, the radial component corresponds to the magnitude of the vector, $\left| P \right|$

|  | $P_{r}=\left\vert P \right\vert=\sqrt{P\cdot P}$ | (S11) |
| --- | --- | --- |

The figure shows that the magnitude of the total dipole (per molecule) is larger in the confined configuration than it is in the bulk. This behaviour may explain why the method used by Martí *et al.*, which is based only on the absolute magnitude of the total cell dipole and ignores any anisotropy, predicted an increase in the dielectric constant of water ^8,9^.


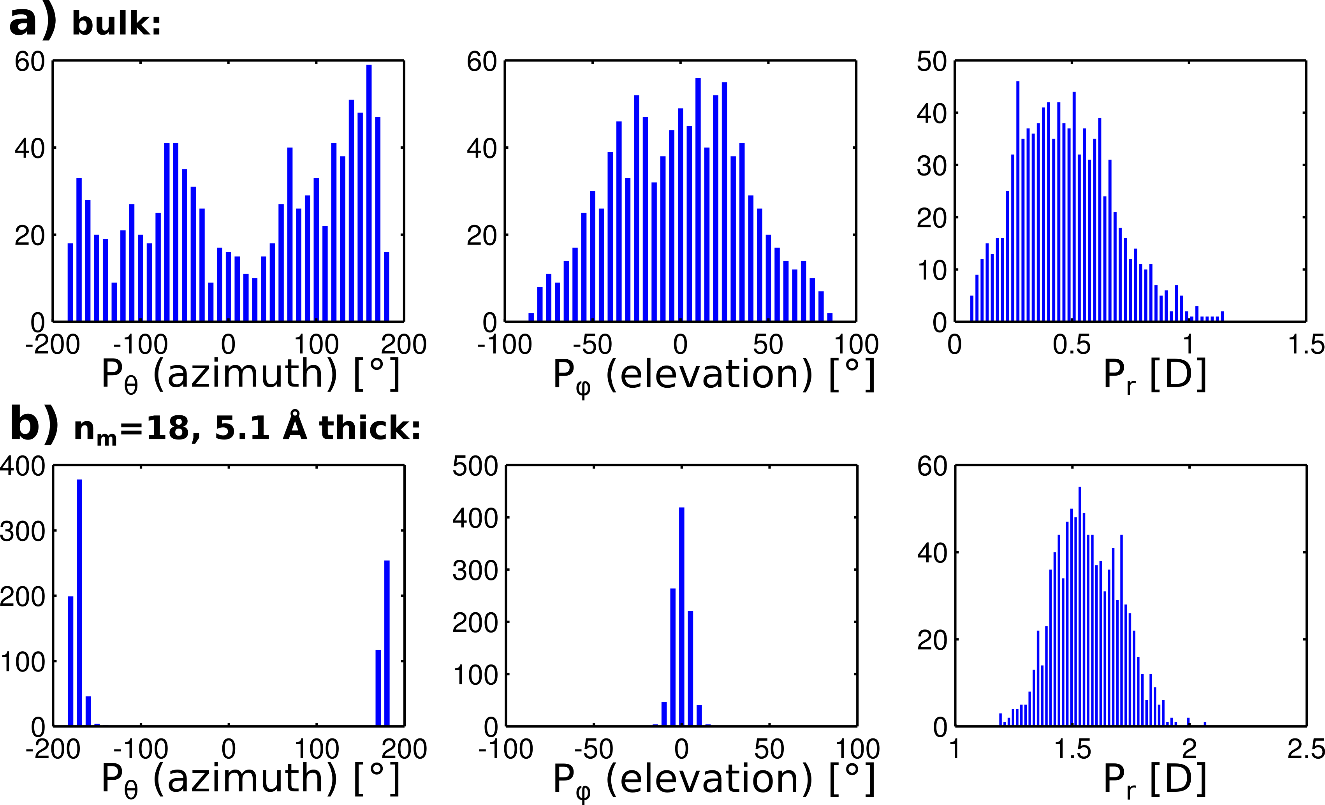


Figure S18. Distribution of the total cell dipoles (per molecule) in spherical coordinates for bulk (a) and a monolayer (b) of NMP.

ADDITIONAL REFERENCES FOR SUPPLEMENTARY INFORMATION

1. Haynes, W. M. *CRC Handbook of Chemistry and Physics*. (CRC Press, 2013).

2. Qiu, J. *et al.* Liquid infiltration into carbon nanotube fibers: effect on structure and electrical properties. *ACS Nano* **7,** 8412–22 (2013).

3. Humphrey, W., Dalke, A. & Schulten, K. VMD: Visual molecular dynamics. *J. Mol. Graph.* **14,** 33–38 (1996).

4. Horn, R. G. & Israelachvili, J. N. Direct measurement of structural forces between two surfaces in a nonpolar liquid. *J. Chem. Phys.* **75,** 1400 (1981).

5. Shih, C. J., Lin, S., Strano, M. S. & Blankschtein, D. Understanding the stabilization of liquid-phase-exfoliated graphene in polar solvents: Molecular dynamics simulations and kinetic theory of colloid aggregation. *J. Am. Chem. Soc.* **132,** 14638–14648 (2010).

6. Müller, G., Lutz, M. & Harder, S. Methyl group conformation-determining intermolecular C–H...O hydrogen bonds: structure of N -methyl-2-pyrrolidone. *Acta Crystallogr. Sect. B Struct. Sci.* **52,** 1014–1022 (1996).

7. Algara-Siller, G. *et al.* Square ice in graphene nanocapillaries. *Nature* **519,** 443–445 (2015).

8. Gordillo, M. C. & Martí, J. Water on graphene surfaces. *J. Phys. Condens. Matter* **22,** 284111 (2010).

9. Martí, J., Sala, J. & Guàrdia, E. Molecular dynamics simulations of water confined in graphene nanochannels: From ambient to supercritical environments. *J. Mol. Liq.* **153,** 72–78 (2010).
